# The Role of Genetic Mutations in Mitochondrial-Driven Cancer Growth in Selected Tumors: Breast and Gynecological Malignancies

**DOI:** 10.3390/life13040996

**Published:** 2023-04-12

**Authors:** Ibolya Czegle, Chelsea Huang, Priscilla Geraldine Soria, Dylan Wesley Purkiss, Andrea Shields, Edina Amalia Wappler-Guzzetta

**Affiliations:** 1 Department of Internal Medicine and Haematology, Semmelweis University, H-1085 Budapest, Hungary; 2 Department of Pathology and Laboratory Medicine, Loma Linda University Health, Loma Linda, CA 92354, USA

**Keywords:** mitochondrial fission/fusion, OXPHOS, mitophagy, *BRCA1/2*, *HER2*, *PTEN*, *ARID1A*, *TERT*, breast cancer, endometrial and ovarian cancers

## Abstract

There is an increasing understanding of the molecular and cytogenetic background of various tumors that helps us better conceptualize the pathogenesis of specific diseases. Additionally, in many cases, these molecular and cytogenetic alterations have diagnostic, prognostic, and/or therapeutic applications that are heavily used in clinical practice. Given that there is always room for improvement in cancer treatments and in cancer patient management, it is important to discover new therapeutic targets for affected individuals. In this review, we discuss mitochondrial changes in breast and gynecological (endometrial and ovarian) cancers. In addition, we review how the frequently altered genes in these diseases (*BRCA1/2*, *HER2*, *PTEN*, *PIK3CA*, *CTNNB1*, *RAS*, *CTNNB1, FGFR*, *TP53*, *ARID1A*, and *TERT*) affect the mitochondria, highlighting the possible associated individual therapeutic targets. With this approach, drugs targeting mitochondrial glucose or fatty acid metabolism, reactive oxygen species production, mitochondrial biogenesis, mtDNA transcription, mitophagy, or cell death pathways could provide further tailored treatment.

## 1. Introduction

The role of mitochondria in solid tumors has been widely investigated in the last decade, along with their contribution to the development and progression of hematologic malignancies [1,2,3,4,5,6,7]. Given the wide spectrum of mitochondrial-related changes existing in cells, which helps them to adapt to new environments, their role in tumorigenesis is also complex. Their altered roles in glucose metabolism, reactive oxygen species production, and apoptosis regulation or the disturbed regulation of mitochondrial fission and fusion (also known as mitochondrial dynamics), mitophagy, and mitochondrial trafficking can all result in the enhanced survival of tumor cells. This survival benefit and better environmental adaptation over normally functioning cells further contributes to chemotherapy resistance [1].

In clinical practice, cancer patients often undergo molecular and cytogenetic studies to determine the appropriate targeted therapy and to provide prognostic data [1]. Our understanding of so-called driver mutations has been expanding, making today’s targeted therapies, such as antibody treatments or cellular therapies, possible. This has subsequently improved patient outcomes.

In this review, we will discuss the common genetic changes seen in breast cancers (BCs), endometrial cancers (ECs), and epithelial ovarian cancers (OCs), as there are overlaps in their pathology, tumorigenesis (see also Section 5), and driver mutations. In addition, we summarize the mitochondrial changes associated with these cancers. Given the clinical significance of their driver mutations, we highlight the mitochondrial changes seen in those gene alterations (*BRCA1/2*, *HER2*, *PTEN*, *PIK3CA*, *CTNNB1*, *RAS*, *CTNNB1, FGFR*, *TP53*, *ARID1A*, and *TERT*). There are many more genes that are associated with the development or progression of these tumors; here, we will only discuss selected genes with their effects on mitochondria. For more details on mitochondria-related cellular metabolism, apoptosis regulation, mitochondrial dynamics, mitophagy, and mitochondrial trafficking, along with information on the mitochondrial DNA (mtDNA) and mtDNA transcription machinery in general, please see our previous article [1]. Various drugs have been proposed for inhibiting metabolic and/or mitochondrial pathways in tumor models and clinical trials, including glycolysis inhibitors (blocking hexokinase 2, phosphofructokinase 2, pyruvate kinase, lactate dehydrogenase A, or pyruvate dehydrogenase kinase) [1,8], oxidative phosphorylation (OXPHOS) inhibitors (via drugs such as metformin, atovaquone, or arsenic acid) [1,9], nucleic acid metabolism inhibitors [10], or inhibitors of abnormally induced fatty acid synthesis, oxidation, or uptake [1,11]. A selection of drugs targeting specific mitochondrial functions, such as mitochondrial fission/fusion, mitochondrial trafficking, and mtDNA transcription or translation, are also discussed in more detail in our previous work on hematologic malignancies [1].

## 2. Breast Cancer (BC)

With its incidence increasing due to population aging, as well as improved detection with more widespread mammography screening, BC has become one of the most common malignancies in women worldwide. Besides other risk factors, such as smoking, long-term estrogen exposure (including hormonal replacement therapy), low parity, high breast density, and ionizing radiation, BC has a significant genetic background in both familial (*BRCA1*, *2*) and sporadic (somatic mutations) cases [12]. A recently emerging topic in the scientific literature is the potential effect of pathological changes in mitochondrial metabolism and mutations in the mitochondrial genome on carcinogenesis. In this review, we explore the link between common BC-related mutations and mitochondrial genetics and mitochondrial metabolism in the pathogenesis of cancer development, which also often contributes to therapy resistance.

The two most common histological types of BC are invasive carcinoma of the breast, not otherwise specified (NOS, previously named ductal carcinoma) (70–75%), and invasive lobular carcinoma (12–15%). The other 18 subtypes exhibit specific morphological traits and are rare (from 0.5% to 5%) [13,14]. BC has distinct pathological subtypes based on the immunohistochemical evaluation of the protein and/or gene expression of ER (estrogen receptor), PR (progesterone receptor), and HER2 receptor, along with the Ki-67 proliferation index [15,16,17,18]. Their distinct pathology is based on their genetic background and leads to different clinical behaviors and responses to various therapeutic interventions (see Table 1, adapted from Goldhirsch and co-workers [19] and Cardoso and co-workers [20]). In Table 1, we list the most common intrinsic BC types, whose possible genetic and mitochondrial backgrounds are discussed in this review [20].

### 2.1. Mitochondria in BC Pathogenesis

**Figure 1:**
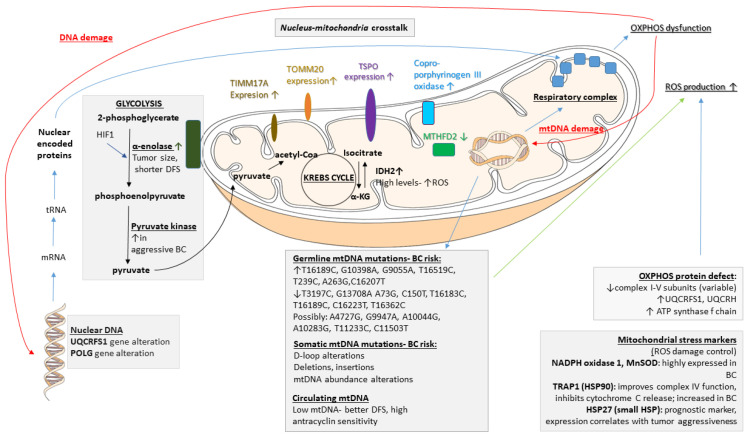
Mitochondrial metabolism, genetics, nuclear–mitochondrial crosstalk, and their roles in BC. There is increased glycolysis in BC. OXPHOS is generally decreased or dysfunctional in most BC cell lines, with the variably decreased expression and/or function of the subunits (or subunit components) of complex I–V. On the contrary, increased complex III subunit (UQCRH and UQCRHS1) expression and increased complex V/ATP synthase f chain expression have also been seen in BC. The asynchrony of complex protein expression jeopardizes ATP production and also leads to increased ROS production. Increased ROS production results in nuclear and mitochondrial DNA (mtDNA) damage, which in turn alters mitochondrial protein (some are encoded by mtDNA, with the majority being encoded by nuclear DNA) expression, including the structural proteins TIMM and TOMM. Interestingly, POLG alterations in BC, encoding for the mtDNA polymerase γ, has been seen in BC. These alterations may result in large-scale deletions and even mtDNA depletion, with the latter compromising OXPHOS and possibly other mitochondrial functions. Additionally, mitochondrial stress markers can be high in BC, and in some cases, their increased expression is a prognostic marker. Additionally, the expression of coproporphyrinogen III oxidase and IDH2 increases, resulting in increased heme synthesis and electron shuttling from the cytosol, respectively. Interestingly, MTHFD2 expression is decreased in BC cell lines, providing nucleotide precursors. Abbreviations: POLG: DNA polymerase γ; UQCRFS1: Ubiquinol-cytochrome c reductase; Rieske iron-sulfur polypeptide 1; UQCRH: Ubiquinol-Cytochrome C Reductase Hinge Protein; DFS: disease-free survival; OS: overall survival; ROS: reactive oxygen species; TRAP1: TNF receptor-associated protein 1; HSP: heat shock protein; IDH: isocitrate dehydrogenase; TIMM: translocase of the inner mitochondrial membrane; TOMM: translocase of the outer mitochondrial membrane; MCF7: Michigan Cancer Foundation-7 cell line; MDA-MB-231: M.D. Anderson-Metastatic Breast 231 cell line; T47D: breast cancer cell line; HIF1: hypoxia-inducible factor 1; MTHFD: methylenetetrahydrofolate dehydrogenase.

Various mitochondrial metabolic pathways have been connected to carcinogenesis: glycolysis, OXPHOS, the tricarboxylic acid cycle (TCA cycle), distinct reactions of the urea cycle, the fatty acid cycle, and gluconeogenesis, which take place in mitochondria. Mutations in mtDNA and alterations in morphological changes (fission/fusion) can contribute to cancer formation as well. In this part of the review, we summarize the metabolic and genetic alterations and their relationships with mitochondria in BC tumorigenesis (see Figure 1) [21].

Anaerobic glycolysis. In cancer cells, pyruvate is abundantly transformed into lactate by anaerobic glycolysis [22], with the overexpression of glycolysis genes generally present [23].

1. *Enolases.* Enolases catalyze the conversion of 2-phosphoglycerate to phosphoenolpyruvate. These enzymes are typically located within the cytosol, yet they tightly associate with the mitochondrial surface [24]. In human tissues, three genetic loci, namely, α, β, and γ, encode the different enolase isoforms. Enolase 1 is present in almost all adult tissues, enolase 2 is found in neuronal and neuroendocrine tissues, and enolase 3 is found mainly in muscle. The enzyme is upregulated under stress conditions via the activation of hypoxia-inducible factor-1 (HIF-1). The overexpression of α-enolase is associated with tumor development, which also serves as a potential diagnostic and prognostic marker [25]. In BC, α-enolase gene expression correlates with tumor size and a shorter disease-free interval [26].
2. *Pyruvate kinase*. Pyruvate kinase (PK) is a rate-limiting glycolytic enzyme that converts phosphoenolpyruvate to pyruvate with the generation of one ATP molecule. It has two isoforms, PKM1 and PKM2, which are encoded by the same gene and are generated by alternative splicing. PKM1 is found mainly in normal cells, whereas PKM2 is an embryonic isoform that is expressed in cancer cells [27]. Elevated levels have been found to be associated with aggressive breast carcinomas [28].

Oxidative phosphorylation (OXPHOS) markers. According to the Warburg hypothesis, the increased rates of anaerobic glycolysis that are observed in tumor cells might be due to their impaired respiratory capacities [22]. Reduced respiration is associated with cancer; however, OXPHOS is not always compromised as a whole. For example, the protein levels or activities of individual OXPHOS enzymes are not uniformly decreased in different BC cell lines. In cell line MCF7, for example, complexes II, III, and V activities and/or levels are decreased, whereas, in cell line T47D, it is complexes I and III that are decreased; in cell line SKBr3, it is complexes III, IV, and V that are decreased; and in cell line MDA-MB-231, it is complexes I, III, IV, and V that are decreased [29]. In line with the Warburg hypothesis, however, the most aggressive BC line displays the broadest OXPHOS defect. In addition to reduced activities [29], complex III activation has also been reported. Complex III subunits UQCRFS1 and UQCRH are overexpressed in a variety of tumors. Increased UQCRFS1 and UQCRH transcription, with increased UQCRFS1 immunoreactivity, was described in BC when compared to normal breast tissue [29]. Additionally, UQCRFS1 gene amplification has been detected in BCs [30]. Regarding complex V, a proteomic study showed a 2-fold increase in the ATP synthase f chain in BC cells [31].

Other metabolic markers. Among the numerous cancer markers in mitochondria, hydratases, dehydrogenases, and oxidases play crucial roles in BC pathogenesis.

1. *Hydratases*. The NAD-dependent bifunctional methylenetetrahydrofolate dehydrogenase/cyclohydrolase (MTHFD) regulates the biosynthesis of tetrahydrofolate, providing precursors for nucleotides and methylation reactions. The MTHFD2 protein content is 3-fold decreased in BC lines [31].
2. *Dehydrogenases.* Isocitrate dehydrogenases (IDHs) are important players in the exchange of metabolites within the cell, and two IDH isoforms can be found within the mitochondrion. IDH2, an NADP-dependent enzyme, has a role in the shuttling of electrons between the mitochondrion and the cytosol. IDH3 is an NAD-dependent mitochondrial matrix enzyme that is involved in the TCA cycle. BC cell lines display high levels of IDH2, and its expression is positively associated with overall survival in BC patients [32], possibly due to enhanced reactive oxygen species (ROS) protection.
3. *Oxidases.* Coproporphyrinogen III oxidase (HemN), an enzyme required for heme synthesis, is present in the inner mitochondrial membrane. Its expression is increased in Adriamycin-resistant BC cells [33].

In Table 2, we summarize the association of mitochondrial metabolic pathways and respiratory chain complexes with BC metabolism.

### 2.2. Mitochondrial DNA (mtDNA) and BC

mtDNA is particularly susceptible to mutations due to its proximity to ROS generation and the relatively inefficient mtDNA repair system [34]. The frequency of mtDNA mutations in cancer cells is 10-fold higher than that of nuclear DNA mutations [35]. Many alterations in mtDNA that can be detected in tumor cells potentially alter mitochondrial function, and mtDNA alterations are often already found in the premalignant stage. Generally, mtDNA is abundant and readily detectable in blood, urine, and saliva samples, making it an attractive subject for diagnostic investigations in many cancer types.

*mtDNA and breast tumorigenesis*. For a great review on this topic, see, for example, Yadav and Chandra [36]. In the last decade, various alterations in mtDNA have been described in BC, including point mutations, mtDNA polymorphisms, mtDNA depletion, microsatellite instability (MSI), insertions, changes in mtDNA copy number, and homoplasmy and heteroplasmy of mtDNA [37,38,39,40,41,42,43,44,45,46,47,48,49,50,51,52,53,54,55,56,57,58,59]. In addition, breast nipple aspirate fluid with different mtDNA mutations (positions 204, 207, and 16293) has been suggested to be an indicator of BC [60]. An mtDNA D-loop mutation has also been proposed as an independent prognostic marker of the disease [61]. Mutations in mtDNA could subsequently involve tRNAs and rRNA [62,63,64], which are required for the synthesis of peptides important in the assembly of various mitochondrial complexes. Therefore, the ultimate outcome of several mtDNA mutations is defective OXPHOS function, which leads to defective aerobic glycolysis and increased ROS production, promoting tumorigenesis [65,66,67]. Altogether, mtDNA instability plays an important role in tumorigenesis, and its most important causes (germline and somatic mutations, displacement loop (D-loop alterations), deletions and insertions, and mtDNA abundance) will be discussed here.

**Figure 2:**
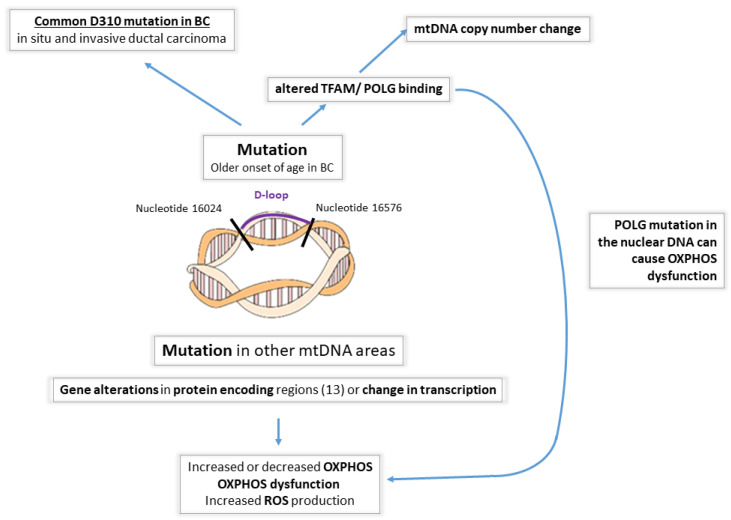
The role of mtDNA D-loop mutations in BC. D-loop mutations, most frequently associated with an older age of onset of BC, can lead to altered mtDNA replication via altered TFAM/POLG binding (these are coded by nuclear DNA), leading to mtDNA copy number and mitochondrial number changes. D310 mutations (replication origin of mtDNA) are frequently seen in association with in situ and invasive ductal carcinomas. Mutations in other areas of mtDNA can alter protein-encoding regions—encoding 13 protein components of the respiratory chain complex proteins—or alterations in the transcription of these proteins can lead to increased or decreased OXPHOS protein expression or the expression of dysfunctional OXPHOS proteins and may induce ROS production. Interestingly, POLG mutations can also cause OXPHOS dysfunction via multiple large-scale deletions and mtDNA depletion. Abbreviations: BC: breast cancer; OXPHOS: oxidative phosphorylation; TFAM: mitochondrial transcription factor A; POLG: DNA polymerase γ.

1. *Germline mtDNA mutations*. BC cells, like other cancer types, commonly harbor instability in the mitochondrial genome [68,69,70,71]. In this section, we discuss some of the widely investigated mtDNA polymorphisms that affect breast carcinogenesis. In the mtDNA T16189C germline mutation, various factors contribute to the substitution of T by C at nucleotide position (np) 16189, which is associated with susceptibility to BC development [72]. The 10398A allele of the NADH dehydrogenase-3 locus (ND3) of mtDNA is associated with an increased risk of invasive BC in African-American women [58,59] and in North Indian women [59]. The 10398G polymorphism of ND3 has been shown to increase the risk of BC in European American, Polish, and Malay populations [45,55,59,73,74]. It is also possible that polymorphisms in the mitochondrial genome could interact with life style and nutritional factors, such as alcohol consumption [75]. Chronic alcohol use may cause OXPHOS deficiency and other cellular changes. The mechanism by which the presence of these mutations leads to mitochondrial dysfunction is not clearly defined, but the G10398A variant of mtDNA may result in defective complex I function and thus lead to increased ROS production [59,76]. Whether ROS produced due to the G10398A polymorphism are sufficient to induce tumor formation remains to be determined, but the presence of other mutations combined with G10398A may contribute to breast tumorigenesis. Other single-nucleotide polymorphisms (SNPs) in mtDNA, including G9055A, T16519C, T239C, A263G, and C16207T, may also result in increased susceptibility to BC [45,73]. mtDNA T3197C and G13708A SNPs decrease the BC risk [73], and reduced incidences of mtDNA A73G, C150T, T16183C, T16189C, C16223T, and T16362C SNPs were noted in BC patients compared to database controls [46], along with other mtDNA polymorphisms associated with BC [77]. An analysis of the sequences of genes encoding complex I in cancer tissues and corresponding normal tissues led to the discovery of very rare mtDNA polymorphisms, including A4727G, G9947A, A10044G, A10283G, T11233C, and C11503T, that may have implications in BC development [46].
2. *Somatic mtDNA alterations.* Despite the fact that numerous germline mutations have been linked to breast tumorigenesis, the majority of BCs are not inherited. In sporadic BC cases, somatic mtDNA mutations may lead to the selective transformation of breast epithelial cells and tumorigenesis. Various somatic mtDNA mutations have been detected in BC [39,42,50,61,78,79,80,81,82,83,84,85,86]. The majority of somatic mtDNA mutations occur in the D-loop region and can be point mutations, deletions, insertions, or missense mutations.
3. *mtDNA displacement loop alterations* (Figure 2). The D-loop is considered a hot spot for mutations [79] and is up to ~60 times more susceptible to mutations than the coding regions, according to some studies. The increase in susceptibility, however, is variable among different studies, with some showing only a 7-fold increase [60]. The D-loop itself is a noncoding region, but mutations in this area are typically significant and potentially affect the expression of mtDNA-encoded protein/s or alter mtDNA replication. The replication of mtDNA starts in the displacement loop (D-loop) region located between nucleotides 16024 and 16576. mtDNA replication involves DNA polymerase γ (POLG) and mitochondrial transcription factor A (TFAM), the latter being the key transcription factor regulating mtDNA copy numbers [87,88]. In BC patients, the occurrence of D-loop mutations is associated with an older age of onset [61]. A homopolymeric C-stretch within the D-loop, termed the 310 microsatellite sequence, is a relatively conserved region that includes the replication origin of the mtDNA heavy strand [89]. Previous reports have shown D310 sequence alterations in human cancers, including ductal in situ carcinomas (68%) and invasive ductal carcinomas (71%) [57]. In another small study, 11 of 18 BCs harbored mtDNA mutations, of which 42% were D310 alterations [39]. Histologically normal breast epithelial cells adjacent to invasive ductal carcinomas that carry D310 mutations may already represent tumor cell clonal expansion [57]. However, these may not be representative of a larger cohort.
4. Deletions. Deletion of 4977 base pairs (ΔmtDNA4977 mutation) has been found in BC tissue, but it was also detected in the surrounding normal breast tissue—indicating either the premalignant state of the tissue exhibiting normal morphology, or representing a clinically non-significant alteration [90,91]. In addition, another research reported conflicting data on the role of ΔmtDNA4977 mutation in BC [61]. Later studies, however, demonstrated that the ΔmtDNA4977 mtDNA deletion, when associated with significant other nuclear gene alterations, such as in the BRCA, ER or TP53 genes, led to premature aging and breast tumorigenesis [92,93].

5. *Alterations in mtDNA abundance*. Mitochondria have multiple copies of mtDNA, and this copy number changes in response to energy demands, with both increased and decreased mtDNA content previously reported in cancer cells [94,95]. In the majority of BCs, the mtDNA content was decreased compared to the adjacent histologically normal tissue when measuring the mean mtDNA content using quantitative RT-PCR and *ND1* gene primers [61].

Interestingly, the circulating mitochondrial nucleic acid copy number could be used as a prognostic marker for BC. Patients with lower mtDNA copy numbers have better disease-free survival than patients with high mtDNA content when treated with anthracycline after surgery [96], likely representing tumor lysis. Additionally, low mtDNA content possibly enhances the sensitization of cancer cells to anticancer agents via altered metabolism or ROS production.

### 2.3. Nuclear DNA Alterations Affecting Mitochondrial Function in Cancer

Nuclear-DNA-encoded proteins are also an integral part of the OXPHOS system, and conversely, defects in OXPHOS induce irreversible changes in the nuclear genome [97,98,99]. Mitochondria–nucleus crosstalk and mitochondrial retrograde signaling play important roles in tumor development [68,100,101,102,103,104]. Additionally, many nuclear-encoded genes have been found to be involved in mitochondrial function [105]. They include, but are not limited to, mutations in genes encoding structural OXPHOS subunits, OXPHOS assembly factors, and components of the mitochondrial protein translation machinery. Nuclear DNA alterations in regions coding the OXPHOS system have not been studied as extensively as mtDNA alterations but are increasingly recognized in cancer. Among respiratory chain proteins, 10 of the 11 structural subunits that make up complex III are encoded by nuclear genes. In BC, the nuclear gene *UQCRFS1,* encoding complex III proteins, can be amplified [30]. DNA polymerase γ (POLG) is responsible for the replication of mtDNA, and mutations cause multiple large-scale deletions and mtDNA depletion, leading to compromised OXPHOS functioning. Mutations in the POLG gene have been detected in BCs and are associated with mtDNA depletion in cancer cells [37].

### 2.4. Mitochondrial Stress Markers in BC

Mitochondrial respiration constitutively produces ROS, and OXPHOS dysfunction further increases their generation. In cancer cells, a further increase in ROS can be observed.

1. *ROS damage control.* NADPH oxidase 1, a major source of ROS in cells, predominantly localizes to the mitochondria and is highly expressed in breast (86%) tumors [102]. To counteract the damaging effects of ROS, cells contain a multilayered system of antioxidant defenses executed by three types of enzymes: superoxide dismutases (SODs), peroxidases (PODs), and catalases (CATs). MnSOD is constitutively present in the mitochondrial matrix, but its expression can be further induced by hypoxia. In BC patients, strong MnSOD staining can be observed in neoplastic cells, with moderate-to-strong staining in adjacent hyperplastic ducts and weak-to-moderate staining in the normal epithelium [106]. A histochemical study shows lower expression in BC cells compared to the adjacent normal epithelia [107].
2. *HSP90 family.* Members of the HSP90 gene family are considered essential regulators of protein folding. TNF receptor-associated protein 1 (TRAP1) is a member of the HSP90 family and is considered mostly mitochondrial. In vivo studies in rats have shown that TRAP1 protects against hypoxia by reducing the generation of ROS, improving mitochondrial complex IV activity, and preserving ATP levels [108]. TRAP1 expression is induced in tumor cells. As shown by immunohistochemistry (IHC), TRAP1 staining appears intense in breast adenocarcinomas, while the normal matched epithelia stain weakly [109]. There is also evidence pointing to the anti-apoptotic role of the HSP90 family. TRAP1 and HSP90 are involved in the mitochondrial pathway that antagonizes the proapoptotic activity of cyclophilin D [109]. This interaction occurs in a multichaperone complex that is selectively assembled in tumor cells and is not present in normal mitochondria [110]. TRAP1 has also been shown to directly interact with members of the MPTP, inhibiting its opening and the subsequent release of cytochrome c (CytC) [111].
3. *Small HSP family.* HSP27 is mainly cytosolic, but a small fraction localizes to the mitochondria. HSP27 expression may function as a useful prognostic marker of poor survival in many human cancers. HSP27 is upregulated in the serum of BC patients [112] and correlates with poor clinical outcomes. A clinical evaluation of BC patients showed the correlated expression of HSP27 with tumor aggressiveness and decreased survival [113].

### 2.5. Mitochondrial Membrane Markers and BC

Mitochondrial function is generally dependent on the import of cytosolic proteins. Complex protein structures form channels that translocate preproteins from the cytosol to the mitochondrial matrix. The proteins that constitute these channels are the translocase of the outer mitochondrial membrane (TOMM) and translocase of the inner mitochondrial membrane (TIMM) [114]. Interestingly, a special outer membrane channel, TOMM20, selectively stains metastatic BC cells but is largely absent from the adjacent lymph node stroma when performing immunohistochemical analysis [115]. A specific inner membrane channel is TIMM17A in BC: proteomic analysis shows a 5-fold increase in TIMM17A protein levels in BC cells, and it shows strong staining in ductal carcinoma in situ and invasive ductal carcinoma of the breast, while the adjacent normal epithelia and stromal cells are negative. All normal breast tissues are TIMM17A-negative; however, elevated protein levels in BC can be detected by IHC and Western blotting as well. Quantitative RT-PCR confirms significantly higher levels in invasive carcinoma compared to normal breast tissue [116]. In line with these findings, a recent study reported the upregulation of TIMM17A mRNA in BC [117]. Both studies have shown that TIMM17A expression is associated with poorer disease-free and overall survival, with TIMM17A therefore being a promising diagnostic and possible prognostic marker for BC patients.

Translocator protein (TSPO), also known as peripheral-type benzodiazepine receptor, is a well-conserved protein located at OMM-IMM contact sites and is closely associated with VDAC and ANT. TSPO has been shown to participate in apoptotic processes but has been described as having both anti- and proapoptotic properties. The overexpression of TSPO is associated with aggressive tumor subtypes in breast carcinomas and correlates with advanced stages of malignancy. Metastatic breast adenocarcinomas manifest increased TSPO expression relative to their primary malignancies [118].

Increasing evidence shows the involvement of mitochondrial dynamics in cancer development, but structural mitochondrial alterations appear to be heterogeneous and nonspecific to neoplasias. There is an increased mitochondrial mass in BC [119], with a markedly increased rate of mitochondria with damaged cristae structures in vitro [120]. Furthermore, altering mitochondrial dynamics, such as fission and/or fusion, helps tumor cells to adjust their bioenergetics and biosynthetic needs. This allows them to be more adaptable to survive in harsh conditions and supports tumor progression. In addition, it is strongly related to apoptosis regulation in most cells (for more details on this topic, see the publication by Avagliano and co-workers, 2019 [121], or Czegle and co-workers, 2021 [1]). In BC, the upregulation of fission protein dynamin-related protein 1 (Drp1) is associated with enhanced glycolysis and mitophagy. The reduction in mitochondrial number due to mitophagy is reversed by an increase in mitochondrial biogenesis. Under low-nutrient conditions, BC cells actually tend to have a fusion predominance and have hyperfused mitochondria by inhibiting Drp1 and favoring energy production through OXPHOS [121,122,123,124].

### 2.6. Genetic Background and Mitochondria in BC

Besides environmental factors, many genetic settings (intrinsic factors) have been proven to drive BC initiation and progression (for a review, see [125]). Similar to other malignancies, the activation of oncogenes and the deactivation of tumor suppressor genes (TSGs) play a role in the modification of cell function, leading to tumorigenesis [126]. Although not all TSGs are vulnerable to mutations, other genetic mechanisms can indirectly interrupt their expression, modifying their functions to induce tumorigenesis [127]. Several genes, such as *TP53*, *BRCA1*, *BRCA2*, *PTEN*, *ATM*, *CDKN1B* (coding protein p27), *SKP2*, and *RAD51*, are well-known TSGs involved in DNA repair and other cellular mechanisms [128,129]. They are further classified into gatekeepers or caretakers based on their functions. Caretaker genes are mainly involved in the healthy function of cells by encoding products that stabilize the entire genome and protect genes from mutational events, such as *BRCA1* and *BRCA2* genes (on chromosomes 17q21 and 13q12, respectively), widely known genetic markers of hereditary breast cancer (HBC). Based on their functions, other similar genes were suspected, and some were later proven, to act as predisposing factors in BC [130,131,132]. Increasing data on *BRCA1*/*2* gene function in the DNA damage response pathway eventually led to the identification of a discrete number of susceptibility genes, including *ATM*, *BRIP1*, *CASP8*, *CHEK2*, *NBN*, *PALB2*, *PTEN*, *TP53*, and *STK11* [130,132,133,134,135,136,137,138].

Besides tumor suppressors, housekeeping genes, such as *PUM1*, *B2M*, *ACTB*, *RPL13A*, *LDHA*, and *NONO*, regulate basic cellular functions governing or preventing cell growth. Their mutations therefore promote cell proliferation [139]. In BC, as in other cancer types, a set of significant gene mutations (somatic and germline mutations) are strongly associated with tumorigenesis by giving cell survival and growth advantages to cancerous cells; thus, they are also known as driver mutations [140]. Most of the driver mutations occur at the somatic level, while a small number of mutations are passed down at the germline level (5–10%), with the latter causing different types of familial BCs [141]. Besides the previously mentioned genes, other driver gene mutations in breast cancers include *AKT1*, *GATA3*, *PIK3CA*, and *MAP3K1* [142,143,144,145]. In addition, mutations in *CBFB* and *RUNX1* have also been described among somatic mutations in BC. Deletion or translocation events in tumor suppressor genes, such as *AKT3* and *MAGI3*, have also been associated with breast tumorigenesis. Recent studies on BC driver genes uncovered an additional list of genes involved in tumorigenesis, including *CCND1*, *ERBB2*, *FGFR1*, *MYC*, *PIK3CA*, *PTEN*, *GATA3*, *MAP3K1*, and *RB1* [125,146,147].

In this part of the review, we summarize the participation of selected well-known and common somatic mutations that influence breast tumorigenesis via mitochondria and mitochondrial metabolism. In addition, some of the genes involved in breast tumorigenesis are also important in EC and/or OC and will be discussed in those sections.

#### 2.6.1. BRCA1

**Figure 3:**
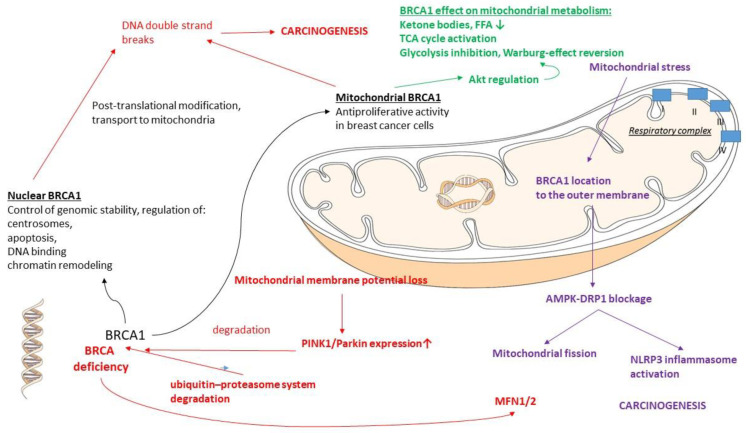
The role of nuclear and mitochondrial BRCA1 in breast carcinogenesis. PINK1: PTEN-induced putative kinase 1; FFA: free fatty acid; TCA cycle: tricarboxylic acid cycle; AKT: alpha serine/threonine protein kinase B; AMPK: AMP-activated protein kinase B; NLRP3: NLR family pyrin domain-containing 3; Drp1: dynamin-related protein 1; Mfn1,2: mitofusin 1,2.

The *BRCA1* gene, when harboring germline mutations, confers a high susceptibility to breast and ovarian cancer predisposition and may account for a total of 10% of the BC incidence [148] (Figure 3). The main role of the BRCA1 protein is the control of genomic stability in the nucleus. BRCA1 is also involved in cell cycle regulation and checkpoint activation [148,149] by modulating specific transcriptional pathways and many highly specialized DNA repair processes [150,151]. BRCA1 is also implicated in the regulation of centrosomes, apoptosis, DNA binding, and chromatin remodeling [152,153]. With current advanced molecular technologies, a large number of mutations in the *BRCA1*/*2* genes have been found in individuals with a family history of BC [154,155]. Pathogenic mutations in the *BRCA1*/*2* genes, however, account only for ~40% of familial BC cases, with a wide cohort of subjects harboring wild-type *BRCA1*/*2* genes [93].

It has been demonstrated that the majority of mitochondrial proteins are nuclear encoded and post-translationally imported in the mitochondria [156]. The nuclear, cytoplasmic, and mitochondrial localization of BRCA1 proteins in human cells was recently evidenced [157], with mitochondrial BRCA1 proteins having an antiproliferative effect on BC cells [158].

DNA double-strand break repair by homologous recombination (HR) is one of the primary mechanisms by which *BRCA1* works as a tumor suppressor. In tumors that lack *BRCA1* function, elevated DNA instability confers sensitivity to poly (ADP-ribose) polymerase (PARP) inhibitors of single-strand break repair, compensating for the lack of HR [159,160]. Additionally, several lines of evidence show that *BRCA1* expression is regulated by the ubiquitin–proteasome system, which involves several E3 ubiquitin ligases, including HERC2, HUWE1, and FBXO44 [152,161,162,163,164,165]. In addition, BARD1 protects BRCA1 from ubiquitin–proteasome degradation by preventing HERC2 from binding the N-terminal degron domain in BRCA1, leading to higher nuclear expression [152]. PINK1 (PARK6) and Parkin (PRKN and PARK2) are key components for mediating the quality control of mitochondria [166]. When mitochondria lose their membrane potential, triggering mitophagy, PTEN-induced putative kinase 1 (PINK1) is stabilized on the mitochondrial outer membrane (MOM). Subsequently, PINK1 phosphorylates both the E3 ligase Parkin and ubiquitin [167,168,169,170,171,172], which finally induces the ubiquitination of MOM proteins, promoting the engulfment of depolarized mitochondria by autophagosomes, also called mitophagy [1,173]. Defective mitophagy is thought to contribute to a variety of diseases, including cancer [1,174]. *BRCA1* is degraded in response to the loss of mitochondrial membrane potential. This proteasomal degradation is dependent on PINK1 and partly mediated through the E3 ligase Parkin. This *BRCA1* degradation causes DNA double-strand breaks. Strikingly, BRCA1 and PINK1/Parkin expression are inversely correlated in cancerous mammary glands from BC patients. *BRCA1* knockdown represses cancer cell growth, and high *BRCA1* expression predicted poor relapse-free survival in BC patients. These observations indicate a novel mechanism by which mitochondrial damage is transmitted to the nucleus, leading to BRCA1 degradation [175].

Coene and co-workers investigated the localization of the BRCA1 protein, known to be involved in nuclear repair pathways. Their confocal and immunoelectron microscopy studies showed that BRCA1 is present in the mitochondria of several human cancer cell lines and in primary breast epithelial cells. Moreover, using small interfering RNA-mediated knockdown of *BRCA1* in human cancer cells resulted in decreased nuclear, cytoplasmic, and mitochondrial BRCA1 presence. In cell fractionation experiments, HeLa cells showed that BRCA1 was enriched in mitochondrial and nuclear fractions but reduced in cytoplasmic subcellular fractions. In addition, the submitochondrial fractionation of rat liver tissue confirmed the presence of BRCA1 in isolated mitoplasts, with electron microscopy studies showing that BRCA1 was localized in the mitochondrial matrix along with mtDNA. Importantly, they found that both nuclear and mitochondrial BRCA1 proteins were hyperphosphorylated, which has been implicated in DNA damage. Taken together, changes in subcellular brca1 localization and phosphorylation are associated with DNA damage, supporting the universal role of BRCA1 in the maintenance of genome integrity in both the mitochondria and nucleus [157].

Mitochondria are dynamic organelles, constantly undergoing fission and fusion, which are essential in mitophagy regulation and the metabolic adaptation of the cells under different circumstances [1,176]. *BRCA1* maintains a healthy mitochondrial network by regulating mitochondrial dynamics, including fission and fusion. *BRCA1* deficiency causes dysfunctional mitochondrial dynamics through the increased expression of the fusion proteins mitofusin 1/2 [177]. During mitochondrial stress, BRCA1 is recruited to the mitochondrial outer membrane, where it plays an essential role in maintaining a healthy mitochondrial network. Consequently, BRCA1 deficiency impairs stress-induced mitophagy by blocking ataxia-telangiectasia mutated (ATM)-AMP-activated protein kinase (AMPK) and dynamin-related protein 1 (Drp1)-mediated mitochondrial fission, triggering NLRP3 inflammasome activation and changing the microenvironment to facilitate tumor proliferation and metastasis [178].

The contributions of *BRCA1* to the metabolic features of cancer cells were investigated by Privat and co-workers. They performed global transcriptional and metabolite profiling of a *BRCA1*-mutated BC cell line with or without transfection with wild-type *BRCA1* in order to obtain a comprehensive view of the participation of *BRCA1* in cancer cell metabolism. The hypermetabolic nature of cancer cells is based on their increased reliance on “anaerobic glycolysis”, namely, the Warburg effect. *BRCA1*, a major tumor suppressor in BC, regulates numerous pathways resulting in anticarcinogenic functions. Their study revealed that *BRCA1* induced numerous modifications in cellular metabolism, including the strong inhibition of glycolysis, along with the activation of the TCA cycle and OXPHOS. The regulation of AKT by BRCA1 in *BRCA1*-mutated breast tumors was suggested to participate in the effect of *BRCA1* on glycolysis. BRCA1 induced a decrease in ketone bodies and free fatty acids, possibly consumed to supply Acetyl-CoA for the TCA cycle. The increased activity of antioxidation pathways was also observed in *BRCA1*-transfected cells, which is likely a consequence of increased ROS production by activated OXPHOS. Globally, the normal function of *BRCA1* in cell metabolic regulation is the reversion of the Warburg effect [179]. See also Table 3.

#### 2.6.2. BRCA2

Oxidative stress is a ubiquitous cellular challenge that generally takes part in carcinogenesis. Pathogenic mutations in the *BRCA2* tumor suppressor gene lead to a general mechanism whereby oxidative stress restricts mtDNA replication. *BRCA2* inactivation induces R-loop accumulation in the mtDNA regulatory region and diminishes mtDNA replication initiation. In *BRCA2*-deficient cells, intracellular ROS are elevated, while ROS scavengers try to save the mtDNA from permanent defects. This molecular mechanism links oxidative stress to mitochondrial dysfunction and is elicited by the inactivation of genes that drive cancer formation [180]. See also Table 3.

#### 2.6.3. ERBB2 (HER2/Neu)

*ERBB2* (also known as *Her2*/*neu*) is an oncogene that is overexpressed in many types of cancers (e.g., breast, ovarian, and gastric cancers), and its activation correlates with a poor prognosis [181]. It was previously demonstrated that, as a driver gene, ErbB2 mutation increases the transformation and/or metastatic potential of human BC [182,183,184,185]. An important effect of ErbB2 is the activation of signaling molecules that regulate bioenergetic metabolism [183,185,186,187,188]. ErbB2 promotes cancer cell growth and glycolysis through the increased expression of lactate dehydrogenase isoform A (LDH-A) [105]. It is well established that ErbB2 localizes to the plasma membrane, where it phosphorylates downstream substrates on their tyrosine residues in response to extracellular stimulation. According to another observation, ErbB2 also localizes to the mitochondria in cancer cells via mtHSP70. Mitochondrial ErbB2 (mtErbB2) negatively regulates mitochondrial respiratory functions, oxygen consumption, and the activities of complexes of the mitochondrial electron transport chain (ETC) in mtErbB2-overexpressing cells. The mitochondrial membrane potential and the cellular ATP level were also decreased. In contrast, mtErbB2 enhances cellular glycolysis. The translocation of ErbB2 and its impact on mitochondrial function are kinase-dependent. Interestingly, cancer cells with higher levels of mtErbB2 were more resistant to the ErbB2-targeting antibody trastuzumab. This finding highlights that mtErbB2 plays an important role in the regulation of cellular metabolism and cancer cell resistance to therapeutics [189]. See also Table 3.

**Table 3:** The effects of BC driver mutations on mitochondrial metabolism.

Driver Gene	Effects on Mitochondrial Metabolism
*BRCA1*	Warburg effect reversal (glycolysis inhibition) [179] Activation of TCA cycle [179] Activation of OXPHOS [179] Mitochondrial BRCA1: antiproliferative activity [158]
*BRCA2*	Mutation causes elevation of intracellular ROS production; oxidative stress causes mitochondrial dysfunction [180]
*ErbB2 (HER2/Neu)*	Promotes cancer cell growth and glycolysis [105,189] Mitochondrial ErbB2: enhances cellular glycolysis [189]

#### 2.6.4. PTEN

Phosphatase and tensin homolog (*PTEN*) will be discussed in more detail in Section 3.2. Here, we just briefly mention *PTEN*, given its relevance in BC, as a driver gene [125,146,147]. Interestingly, a 2018 in vitro BC study showed that chemically modified (CH3- and NH2-modified) hydrophobic surfaces could induce mitochondria-mediated apoptosis by suppressing PTEN, which can be relevant in BC tumorigenesis via its extracellular matrix interactions [190]. See also Table 3.

## 3. Endometrial Cancer (EC)

Endometrial carcinoma, the most common type of uterine cancer (>90%), is a malignant epithelial neoplasm originating from the endometrium. Based on its morphology, it can be further divided into different histologic types, such as endometrioid carcinoma, clear-cell carcinoma, serous carcinoma, carcinosarcoma, squamous cell carcinoma, undifferentiated carcinoma, dedifferentiated carcinoma, and others [191].

*Histology*. Endometrioid EC is the most common type of adenocarcinoma, which has glandular growth with variable architectural patterns, with areas of possible squamous or other differentiation. Grading is based on its architectural complexity, dividing it into low-grade (grades 1–2) and high-grade (grade 3) tumors. The pathophysiology of this type of carcinoma is driven by estrogen that is unopposed by progesterone or progestin. This unopposed estrogen alters the transcription profile of epithelial cells via estrogen receptors (ERs) that directly bind to DNA (see also Section 5 for other estrogen-related effects). Although the exact mechanism of ER in EC tumorigenesis is unknown, it leads to neoplasia formation, preceded by hyperplastic changes [6,192]. Endometrioid ECs are also referred to as type 1 ECs, which are hormonally driven, and generally have a favorable outcome with a strong initial response (~75%) to progestin treatment [192]. The molecular and cytogenetic changes in this group are heterogeneous and include alterations in *PTEN*, *ARID1A*, *KRAS*, *CTNNB*, or *PI3K* genes [193].

As opposed to type 1 ECs, type 2 ECs are non-hormonally driven and are typically higher-grade tumors [192]. Type 2 ECs, such as serous and clear-cell carcinomas, are commonly associated with *TP53* mutations, and their 5-year survival rates are significantly poorer than those of type 1 ECs, even when compared to high-grade endometrioid ECs. Although serous carcinomas frequently arise from endometrial atrophy, the pathophysiology of non-endometrioid cancer formation is not well understood [193,194].

*Genetic background*. In ECs, as in many other tumors, genetic predisposition is also an important factor in carcinogenesis. A familial risk of developing EC has been found in women with Lynch syndrome, also known as hereditary nonpolyposis colorectal cancer syndrome. Inherited germline mutations in *MMR* genes cause defects in mismatch repair proteins, including MLH1, MSH2, MSH6, PMS1, and PMS2, making up ~3% of all EC cases [193,195]. In addition, patients with familial site-specific endometrial carcinoma—a term designated for patients with the clustering of endometrial carcinomas alone with no other cancers—may show germline mutations in *MMR* genes but with lower mutation rates than is seen in Lynch syndrome [195,196]. A higher incidence of EC is also associated with Cowden syndrome, which is characterized by *PTEN* mutations. Additionally, *BRCA1*/*2* pathogenic variants possibly slightly increase the risk of developing *TP53*-mutated serous EC; however, there is currently no consensus on this matter [193].

*Molecular classification.* Recently, in addition to the classic histological typing of ECs, a molecular classification has also been proposed based on The Cancer Genome Atlas (TCGA) classification. This classification divides these tumors into four molecular subtypes [191,197]:

1. Ultramutated (polymerase ε (POLE) mutant): mostly composed of endometrioid ECs, which, despite having an increased mutation frequency and hotspot mutations in the *POLE* gene (encoding the central catalytic subunit of DNA polymerase epsilon), have a better prognosis than other groups.
2. Hypermutated (mismatch-repair-deficient (MMRd)): Involves germline and somatic mutations, resulting in microsatellite instability, such as via *MLH1* silencing due to hypermethylation. In general, tumors in this group are associated with intermediate, stage-dependent prognosis.
3. Copy number high (p53-abnormal): *TP53* alterations are present, with ~50% of cases being serous carcinomas and carcinosarcomas, and ~25% of cases are higher-grade endometrioid ECs. In general, tumors in this group are associated with inferior survival.
4. Copy number low (no specific molecular profile (NSMP)): This group includes *TP53* and *POLE* wild-type and MMR-proficient tumors, which frequently harbor *PTEN*, *PIK3CA*, *ARID1A*, or *KRAS* alterations. The majority of these tumors are low-grade endometrioid ECs [191].

For recurrence-free survival, *PLOE*-mutated and MMR-deficient groups have been shown to have better outcomes, even in the grade 3 EC group [198]. In addition to the gene alterations above, other genes, such as fibroblast growth factor receptors (FGFRs), may also contribute to tumorigenesis in EC, with the tumors harboring *FGFR2* mutations exhibiting poorer outcomes [199]. Current therapies are discussed in other articles in detail [193,198,200,201,202].

Here, we discuss EC and its common genetic alterations in relation to mitochondria.

### 3.1. EC and Mitochondrial Changes

*Changes in OXPHOS.* Many mitochondrial and metabolic changes have been identified in EC, with several attributed to estrogen effects in type I ECs. EC is thought to be a “high OXPHOS” cancer, meaning that, unlike many other tumors showing mostly increased glycolysis, EC heavily relies on OXPHOS [203]. This was shown primarily in *TP53*-deficient EC, which is associated with a poor prognosis [204].

In vitro and in vivo MMRd-deficient EC models revealed impaired mitochondrial function, decreased basal oxygen consumption, and the decreased expression of 32 genes related to electron transport chain function [205]. In type I EC, decreased mitochondrial respiratory chain complex I immunoreactivity was seen in a human study, with a generally increased mitochondrial mass in those tumors compared to normal endometrial tissue. Interestingly, these areas often showed oncocytic changes on H&E [206]. Based on these data, reduced OXPHOS, or complex I expression in some ECs, may contribute to the less aggressive nature of these tumors. Interestingly, a recent report showed that a certain OXPHOS signature (including increased *ATP5IFE* expression, coding a subunit of complex V) in EC cells is associated with better disease outcomes, a lower grade, and a lower stage of EC, along with endometrioid histology [207], prompting further studies of OXPHOS and EC outcomes.

*Anaerobic glycolysis.* In addition, increased anaerobic glycolysis (with increased hexokinase 2, phosphoglucose isomerase, pyruvate kinase/PKM2, and lactate dehydrogenase expression) has been reported in EC, with multiple reports linking the glycolysis-associated gene or long noncoding RNA signature to poorer EC patient outcomes [203]. In addition, EC cells show increased GLUT6 expression compared to normal glands, which helps to support aerobic and anaerobic glycolysis with glucose. In a preclinical EC study, the inhibition of GLUT6 resulted in reduced glycolysis and cell survival, making it a potential tumor therapy candidate [203,208].

*mtDNA copy number changes.* An increase in mitochondrial mass and a two-fold increase in the mtDNA copy number have been identified in endometrioid EC. Serous EC was also found to have an increased mtDNA copy number in a previous study [209]. Interestingly, another study showed an increased mtDNA copy number in EC, with no difference between type I and type II ECs [210]. Enhanced mitochondrial biogenesis is typically an adaptive advantage in which proliferating cells meet the increased energy demand [211]; however, one study failed to show increased mitochondrial biogenesis in EC tissue (n = 148) using PGC-1α and VDAC immunostaining when compared to the normal endometrium. This study, however, included both type I and type II ECs [212]. In addition to increased mitochondrial biogenesis, Drp1-mediated mitochondrial fission, BNIP3-mediated mitophagy, and proteolysis were also found to be enhanced in type I EC [6,213].

*mtDNA mutations.* Both somatic and germline mutations in mtDNA have been identified in the early (~9%) and late (~12%) stages of ECs, with some mutations being tumorigenic and some being adaptive [1,62,214].

*Germline mtDNA mutations.* Germline mtDNA mutations in EC frequently involve the D-loop region, with point mutations such as m.16189t>C commonly identified in endometrioid EC [215]. In addition, D-loop polymorphisms were described in the Polish population (m.16223C>A, m.207G>A, and m.16126T>C), which were found to be associated with an increased risk of EC development in a small study [3]. Additionally, mtDNA mutations outside the D-loop area involving *ND2* (m5178A>C), coding the NADH dehydrogenase 1 enzyme [216], or *COI* (m.7028C>T), coding cytochrome oxidase subunit I [3], have also been associated with increased susceptibility to EC development.

*Somatic DNA mutations.* As for somatic mtDNA mutations in EC, several studies have shown polymorphisms in the D-loop area, in the rRNA-coding area, and in one of the mitochondrial complex protein-coding areas—summarized in an excellent recent review article [217]. Interestingly, estrogen has been associated with an increased number of mtDNA mutations despite its anti-inflammatory effect in other studies, likely due to enhanced mitochondrial biogenesis, which leads to a larger number of replication errors [6].

### 3.2. PTEN

*PTEN in healthy tissues and tumorigenesis.* The phosphatase and tensin homolog (*PTEN*) tumor suppressor gene is found in most human tissues. It regulates transcription, translation, and cell cycle progression, among other processes. In short, it prevents cells from proliferating too rapidly or in an uncontrolled manner. In both heritable and sporadic cancers, the loss of function of PTEN results in genomic instability, cell proliferation and growth, increased cell survival, defective DNA repair, cell migration and adhesion, angiogenesis, and cellular metabolism. Its main downstream target is the PI3K/AKT/mTOR pathway; however, it has other non-enzymatic effects (see later in this section) [218,219,220,221].

The PTEN protein has both lipid phosphatase and protein phosphatase activities. The lipid phosphatase activity is associated with the arrest of G1/S cell cycle progression, whereas the protein phosphatase activity is associated with growth-factor-stimulated MAPK signaling inhibition, cellular adhesion, spreading, and migration. The combined losses of both lipid and protein phosphatase activities result in tumorigenesis with cellular senescence, apoptosis escape, aberrant cell growth, and increased cell spreading and migration [218]. PTEN’s main proapoptotic function is thought to be via the inhibition of the phosphoinositide-3 kinase (PI3K) pathway. As previously mentioned, this is through its lipid phosphatase activity, where it dephosphorylates phosphatidylinositol (3,4,5)-trisphosphate (PIP3) to inhibit AKT and its downstream signaling pathway (see Section 3.3. for the PI3K/AKT/mTOR pathway), altering apoptosis. Thus, *PTEN* deletion or inactivation acts as a positive regulator of the PI3K/AKT/mTOR pathway, mainly via PIP3 accumulation due to a lack of dephosphorylation, which ultimately inhibits apoptosis [222].

Besides its phosphatase activities, PTEN also has non-enzymatic roles: it acts as a scaffolding protein in the nucleus and in the cytoplasm, with the protein having a tumor suppressor function when present in the nucleus. The various PI3K-dependent and PI3K-independent downstream effects make PTEN a major regulator of cell survival and function [218,221,223,224].

*The role of PTEN in mitochondria*. To maintain genomic stability, PTEN acts through its phosphatase-independent (non-enzymatic) function as a scaffolding protein [225]. In addition, PTEN is a crucial mediator of mitochondrial-dependent apoptosis via mitochondrial accumulation and association with the proapoptotic bax protein and the regulation of cellular ROS production [226]. Additionally, previous studies have shown that PTEN is a positive regulator of autophagy via its inhibitory effects on the PI3K–AKT–mTOR signaling pathway, as well as other pathways. It increased autophagic flux and lysosomal mass in a hepatocellular carcinoma cell line [227,228,229,230]. In addition, *PTEN* overexpression was shown to inhibit mitophagy via blockage of the Toll-like receptor 4 (TLR4)–c-JUN N-terminal kinase (JNK)–BCL2/adenovirus E1B 19 kDa protein-interacting protein 3 (BNIP3) pathway. In addition, PTEN-long (PTEN-L), a PTEN isoform, when located at the outer mitochondrial membrane, dephosphorylates ubiquitin to inhibit mitophagy. Moreover, cytosolic PTEN can also suppress mitophagy by targeting the fusion protein Mfn2 and Rab7A (involved in endocytosis). PTEN has a complex role in mitophagy and may be influenced by the microenvironment, the cellular state, and even the cell type [231,232].

*PTEN loss and cellular metabolism*. The effect of PTEN loss on cellular metabolism is also complex, ultimately leading to improved insulin sensitivity, providing a survival advantage for tumor cells through an improved ability to handle metabolic stress. These effects are either via PI3K/AKT signaling or are independent of it. It has been shown that PTEN loss leads to the increased phosphorylation of certain mitochondrial (voltage-dependent anion channel, VDAC) and glycolytic proteins (hexokinase II/HKII, glucokinase/GK, and phosphofructokinase/PFK), leading to increased glycolysis. Additionally, gluconeogenesis is inhibited by PTEN loss via AKT induction, which results in the inhibition of the two rate-limiting enzymes, glucose-6-phosphatase (G6Pase) and phosphoenolpyruvate carboxykinase (PEPCK). Cellular respiration is induced by the upregulation of PI3K/AKT due to its regulation of ERR and NRF, two mitochondrial biogenesis regulators. Moreover, AKT mobilizes the glucose transporter GLUT4 to the cell membrane, which is normally regulated and inhibited by PTEN [233]. PTEN loss has also been shown to increase mitochondrial biogenesis via the induction of mitochondrial transcription factor A (TFAM), mitochondrial transcription factor B1/2 (TFB1M/2M), and peroxisome proliferator-activated receptor-gamma coactivator 1-α (PGC-1α), with the latter increasing both mtDNA replication and transcription [1,233]. Lipogenesis is also induced by PTEN loss via its loss of sterol receptor element-binding protein (SREBP) suppression, the key transcriptional factor involved in fatty acid, triglyceride, and cholesterol synthesis. It binds to several genes involved in lipid synthesis, including fatty acid synthase (FASN), acetyl-CoA carboxylase (ACC), and enzymes controlling NADPH production [233].

*PTEN* inactivation occurs via gene deletions, gene mutations (nonsense, frameshift, splice site mutations, or short in-frame deletions or insertions), and epigenetic or transcriptional alterations [234,235]. In addition, post-translational PTEN protein changes, such as acetylation, oxidation, phosphorylation, sumoylation, or ubiquitination, may result in PTEN instability and the subsequent loss of function [236].

*The role of PTEN in female cancers.* In the female population, *PTEN* has been shown to be one of the most frequently mutated genes (13%) in the four most frequent female cancers: breast, ovarian, endometrial, and cervical cancers [237,238]. The median PTEN alteration rate is 50.54% in EC (38.96% from COSMIC and 62.12% from TGCA), 5.66% in (epithelial) OC (4.98% from COSMIC and 6.34% from TGCA), 8.37% in cervical cancer (4.62% from COSMIC and 12.12% from TGCA), 7.64% in vulva carcinoma (data available only from COSMIC datasets), and 21.05% in uterine carcinosarcoma (data available only from TGCA) [239,240].

*PTEN* mutations can co-exist with other gene mutations and lead to aberrant PI3K/Akt/mTOR pathway activation. For example, the combination of *PTEN* mutations with *KRAS* mutations in the ovary has been shown to induce invasive and widely metastatic endometrioid OC [241].

Several published preclinical models suggest that the loss of *PTEN* in the fallopian tube is one of the multiple genetic modifications that induce tumorigenesis [242]. A recent study showed that the loss of *PTEN* leads to multicellular tumor spheroids in the fallopian tube epithelium. Tumor spheroids are associated with tumor survival under ultra-low-adhesion conditions, tumor attachment to the extracellular matrix exposed during ovulation, and the colonization of tumor cells in the ovary, possibly contributing to high-grade serous OC’s seeding phenomenon in ovaries [243].

In addition, inhibiting glycolysis, lipogenesis, or mitochondrial biogenesis would possibly be additional drug targets in patients with *PTEN*-altered tumors by taking away their metabolic advantage. Inducing mitophagy could also hold therapeutic potential, but more research is required before therapies are going to be available.

See the *PTEN*-related changes summarized in Table 4 [226,227,228,229,230,231,232,233,234,235,236,237,238,239,240,241,242,243,244,245,246,247,248,249,250,251,252,253,254,255,256,257,258,259,260,261,262,263,264,265,266,267,268,269,270,271,272,273,274,275,276,277,278,279,280,281,282,283,284,285,286,287,288,289,290,291,292,293,294,295,296,297,298,299,300,301,302,303,304,305,306,307,308,309,310,311,312,313,314,315,316,317,318,319,320,321,322,323,324,325,326,327,328,329,330,331,332,333,334,335,336,337,338,339,340,341,342,343,344,345,346,347,348,349,350,351,352,353,354,355,356,357,358,359,360,361,362,363,364,365,366,367,368,369,370,371,372,373,374,375,376,377,378,379,380,381,382,383,384,385,386,387,388,389,390,391,392,393].

### 3.3. PIK3CA

*PI3K pathway*. The phosphatidylinositol-3 (PI3) kinase (PI3K) pathway, discovered in the 1980s, consists of a family of lipid kinases that are categorized into three distinct classes (I, II, and III) based on their structure and substrate specificity [244,245]. *PIK3CA* is a proto-oncogene that encodes the class I catalytic subunit (p110α) of PI3K; it is also known as the p110α protein. Its downstream target, the serine/threonine kinase mammalian target of rapamycin (mTOR) enzyme, is the main effector of this pathway. With the increased expression of *PIK3CA*, mTOR is constantly activated, leading to accelerated cell growth, motility, survival, proliferation, protein synthesis, autophagy, intracellular trafficking, and angiogenesis [246,247,248]. The PI3K pathway and its effects are the same as the downstream pathway in *PTEN* mutations; however, *PTEN* mutations also have PI3K-independent alterations (see Section 3.2. for more details). Interestingly, RAS also activates PI3K [247,249]. In addition to genetic alterations, PI3K signaling can also be triggered by other proteins, such as the glucose transporter GLUT1 or hypoxia-inducible factor 1-α (HIF-1α), both of which are often increased in cancer cells [250].

*Metabolic effect of PI3K pathway activation.* The activation of the PI3K/AKT/mTOR pathway results in the previously described Warburg effect (see Section 2.1 and Section 2.6.1). It plays a crucial role in lipid synthesis and glucose metabolism by regulating glycolysis and possibly the TCA cycle [247,250]. The inhibition of the PI3K-AKT-mTOR pathway results in decreased intracellular lipid accumulation with reduced de novo fatty acid synthesis and increased fatty acid oxidation in hepatocytes [251]. Additionally, in patients with EC, decreased PI3K/AKT/mTOR expression was associated with the decreased expression of fatty acid synthase (FASN) and sterol regulatory element-binding protein (SREBP) [252]. Unexpectedly, this was more frequent in higher-grade disease in this small study [252]. Given the small sample size, this would require further investigations with a larger cohort to confirm the findings. Furthermore, the PI3K/AKT/mTOR pathway has an inhibitory effect on the pentose phosphate pathway (via G6PD stabilization) and on PK2, a rate-limiting enzyme of glycolysis. AKT activation also increased glucose uptake and lactate excretion [250]. In addition, previous studies suggested that the PI3K/AKT/mTOR pathway regulates the TCA cycle, although no evidence has yet emerged to prove its direct effect [253].

*PI3K pathway in autophagy and ROS production.* The PI3K pathway is an important regulator of autophagy, depending on the intensity of ROS production. With moderate ROS levels, the PI3K α catalytic subunit is activated, which inhibits autophagy. In contrast, with higher ROS levels, the PI3K β catalytic subunit is activated, promoting autophagy. In addition, AKT also regulates autophagy. With moderate ROS present, AKT inhibits autophagy by inhibiting mTOR complex 1 (mTORC1) and inhibiting the expression of some autophagy proteins (via FoxO transcription factor inhibition). When high levels of ROS are present, however, AKT can induce beclin1-dependent autophagy. Importantly, PTEN is a negative regulator of the PI3K/AKT pathway [254]. In addition, as previously mentioned in Section 3.2, another study found that blocking AKT signaling could inhibit mitophagy [255]. Similarly to *PTEN*, the effect of the PI3K/AKT/mTOR pathway on autophagy is not completely understood.

*PI3K in mitochondria*. Furthermore, inhibiting PI3K and mTOR can block tubulin polymerization, which leads to microtubule disturbances [256], likely inhibiting mitochondrial trafficking. Mitochondria are delivered via microtubules in most cells, including in cancer cells, to the sites where they are needed the most. In invasive cancer cells, in the cytoplasm close to the cell membrane, there is a high ATP demand. This in turn helps to increase cell motility [1]. In contrast to what we would expect, a recent study showed that PX-866, a PI3K inhibitor, paradoxically increases tumor cell motility and “reprograms” mitochondrial trafficking to make them available at the site of invasion [257].

*PI3K/AKT/mTOR pathway in BC, EC, and OC.* The PI3K/AKT/mTOR pathway is one of the most studied intracellular signaling pathways through extensive genomic analysis using molecular profiling by The Cancer Genome Atlas. It helped to identify some of the most common alterations involving metabolic and signaling pathways, especially in OCs (~12%). *PIK3CA* mutations can also be found in BC (~8–40%) and EC (~36% and ~53 endometrioid ECs), among others. In EC, mutations in exon 9, specifically charge-plus changing substitutions, are more likely to lead to the decreased survival of patients with endometrial carcinoma [254]. Mutations in exon 20 have been associated with a higher histological grade and deeper myometrial invasion than exon 9 mutations [258]. Using a different set of data, the median *PIK3CA* alteration rate was found to be 45.42% in endometrial carcinoma, 16.31% in ovarian epithelial tumors, 26.37% in cervical cancers, 19.01% in vulva carcinoma, and 40.35% in uterine carcinosarcoma [239,240,249]. Interestingly, *PIK3CA* mutations have also been shown to drive therapy resistance in epidermal growth factor receptor 2 (EGFR2)-positive BC [259].

Mitochondrial targeting may add to the repertoire of drug selection in PI3K/AKT/mTOR-associated tumors based on their metabolic and other mitochondria-related effects. See the *PIK3CA*-related changes summarized in Table 4.

### 3.4. KRAS

*The role of KRAS in EC. KRAS* gene mutations have been identified in up to 15–30% of endometrioid ECs. *KRAS* is a proto-oncogene that, when mutated, acquires a gain-of-function ability, resulting in increased proliferation, transformation, and cell survival [195]. It belongs to the *RAS* group of proto-oncogenes along with *HRAS* and *NRAS*—discussed in more detail in our previous article, along with their effects on mitochondria [1]. In short, the *KRAS* gene encodes the KRAS protein, which is a signal transductor protein with GTPase activity. The function of KRAS is involved in the regulation of cell division and relies on GTP for activation. Activating mutations typically lead to a conformational change in which the protein is locked in its GTP-bound state, resulting in the robust acceleration of the transcription of a wide range of genes [260,261]. The major downstream pathways of RAS activation include phosphatidylinositol-3-kinase (PI3K), mitogen-activated protein kinase (MAPK), and RAS-like (Ral) small GTPase pathways, resulting in oncogenic changes.

*RAS mutations in mitochondria. RAS* mutations are known to suppress OXPHOS and enhance glycolysis, mostly via STAT3 activation. In addition, the fission protein Drp1 seems to promote tumor growth in RAS-driven pancreatic cancers, whereas Drp1 loss or inhibition impairs mitochondrial metabolism and tumorigenesis [262,263,264]. Conversely, enhanced mitochondrial fusion had a tumor-suppressive effect in a preclinical study [263]. In this work, mitochondrial protein Mfn2 expression was increased by administering doxycycline or leflunomide treatment [263]. In addition, RAS-dependent tumors seem to have high levels of autophagy, and the downregulation of essential autophagy proteins impaired tumor cell growth in an in vitro *RAS*-driven tumor model [265].

Based on the above, inhibiting the metabolic advantage in RAS-driven tumors may improve treatment success, as seen with the use of sotorasib, a KRAS inhibitor that is currently approved for KRAS-mutated non-small-cell lung cancers [266]. In addition, clinical trials showed some success with the inhibition of its downstream pathways [247].

See the *RAS*-related changes summarized in Table 4.

### 3.5. CTNNB1

*CTNNB1 gene*. The *CTNNB1* gene encodes for the β-catenin protein, which is a multifunctional protein and the effector of the Wingless/int1 (Wnt) signaling pathway. Besides this well-documented Wnt/β-catenin pathway, also called the canonical pathway, Wnt has alternative downstream pathways documented in the literature. Although these noncanonical, β-catenin-independent pathways can be associated with carcinogenesis in many cases, here, we only discuss them briefly. In different cancers, the noncanonical pathway includes the activation of various pathways, such as the PCP/Rho/Jun-N-terminal kinase (JUN), receptor tyrosine kinase (RTK) (PI3K/AKT and YAP/TAZ signaling), or Ca2+ signaling cascades activated via the Frizzled ((Fz), as in the canonical pathway), ROR1/ROR2, or RYK receptors. These noncanonical pathways are described in more detail in [267,268].

*The β-catenin signaling (Wnt) pathway*. This is a fairly complex pathway due to the number of participating proteins. Stated simply, β-catenin, when localized in the nucleus, regulates the transcription of many genes, and an increased amount of nuclear β-catenin is present when Wnt is attached to its receptor. This is the so-called “Wnt ON” state. On the other hand, when Wnt is not attached to its receptor, the lack of downstream pathway activation allows β-catenin to be phosphorylated, leading to ubiquitin-mediated proteolysis in the proteosomes. This is the “Wnt OFF” state. Further details on the participating proteins are as follows:

1. “Wnt ON”: Wnt binds to its membrane receptor (the so-called Fz and LRP5/6 receptors) → this induces the cytoplasmic disheveled (DVL) protein, recruited by the Fz receptor → cytoplasmic LRP5/6 protein phosphorylation and Axin protein recruitment → no β-catenin phosphorylation by Axin → no β-catenin degradation → β-catenin accumulates in the nucleus and displaces Groucho/TLE from the TCF-TLE complex, allowing TCF to activate Wnt-responsive genes.
2. “Wnt OFF”: Absent Wnt → no receptor binding and activation → cytoplasmic β-catenin forms a complex with Axin, GSK3, and CK1→ β-catenin phosphorylation by Axin and GSK3 → E3 ubiquitin ligase β-Trcp recognizes phosphorylated β-catenin → β-catenin proteasomal degradation → TCF-TLE complex and histone deacetylases (HDACs) repress Wnt target genes [269,270,271].

*Wnt target genes and their role in carcinogenesis*. Wnt target genes include *MYC* (encoding c-Myc), *CCND1* (encoding cyclin D1), and *CDKN1A* (encoding cyclin-dependent kinase inhibitor p21), among many others. From these genes only, however, it is easy to understand its oncogenic potential. In addition to its effects on cellular proliferation and vasculogenesis, it also regulates cellular development and differentiation, migration, invasion, and general cellular homeostasis [271,272,273]. Although the literature on the Wnt/β-catenin pathway is extensive, here, we only discuss its metabolic and mitochondrial effects in more detail.

*Wnt signaling pathway and mitochondria*. Wnt signaling enhances anaerobic glycolysis in colon cancer cells via increased pyruvate dehydrogenase kinase 1 (PDK1) expression [272,273,274]. PDK1 is a mitochondrial kinase that inhibits the pyruvate kinase complex via pyruvate dehydrogenase (PDH) phosphorylation, resulting in reduced acetyl-CoA production for OXPHOS and enhanced anaerobic glycolysis [275]. Additionally, lactate dehydrogenase A (LDH-A) is indirectly activated by β-catenin via *MYC* and PI3K/Akt/HIF-1α activation [276]. PI3K/Akt/HIF-1α has also been shown to increase glucose transporter activity via β-catenin activation in vitro [277]. In addition, monocarboxylate transporter 1 (MCT1)—a proton-coupled monocarboxylate transporter carrying carboxylate-carrying molecules, such as lactate, pyruvate, and ketones across the cell membrane—is also positively regulated by β-catenin, further improving anaerobic glycolysis and the Warburg effect [272,278]. OXPHOS, however, is often increased along with anaerobic glycolysis in Wnt-driven tumors, although the latter is typically more dominant. OXPHOS-predominant Wnt-driven tumor cells were also found in a previous study, highlighting the heterogeneity of these cells [275,279]. As expected, blocking the Wnt/β-catenin pathway results in decreased anaerobic glycolysis and PDK1 expression, which ultimately has a negative effect on cell growth in vivo [273]. This reduced anaerobic glycolysis, however, was observed to increase again a few days after treatment in an in vitro model, likely via other kinases, highlighting the importance of using additional mitochondrial metabolism blockers (see the list and table in our previous article [1]).

A previous study using a human *CTNNB1*-mutated HCC dataset and an in vivo hepatocellular carcinoma model showed that the upregulation of the Wnt/β-catenin pathway significantly increases fatty acid oxidation [280]. Furthermore, Wnt/β-catenin signaling inhibition results in decreased sterol regulatory element-binding protein-1c (SREBP-1c) expression in hepatocytes, a transcription factor that induces the expression of a family of genes involved in glucose utilization and fatty acid synthesis [281].

The Wnt/β-catenin pathway also regulates mitochondrial dynamics. In hepatocellular cancer cell lines, the inhibition of the Drp1 mitochondrial fission protein leads to mitochondrial dysfunction, energy depletion, and apoptosis induction via Wnt/β-catenin pathway inhibition [282], highlighting the potential benefit of Drp1 inhibition in Wnt-driven cancer cell therapy.

*Wnt/β-catenin pathway and cancer*. As previously mentioned, the Wnt/β-catenin pathway plays an important role in cell fate. In many tumors, the activated Wnt/β-catenin pathway induces apoptosis; however, under specific circumstances, it can induce p53-driven apoptosis. For more details on this topic, see the review article by Trejo-Solis and co-workers (2021) [283].

Activating mutations of the *CTNNB1* gene often lead to a mutated form of β-catenin that is resistant to degradation. The intranuclear β-catenin level is subsequently increased with enhanced gene transcription activity [195]. Altered, predominantly mutant *CTNNB1* genes have been linked to several cancers, including EC (~16–71%) and OC (endometrioid OC ~43%; clear-cell OC ~ 10%), among others [195,284,285]. Gene amplification and gene fusion have also been seen [313,314,315,316]. In ECs, *CTNNB1* mutations have been associated with lower-grade (grades 1–2) disease and early-stage (stages I-II) disease, but also with worse recurrence-free survival [286].

Numerous Wnt/β-catenin-targeting drugs have been researched [2,267,271,287], with mitochondrial targeting possibly enhancing the success of Wnt-driven tumor therapy.

See the *CTNNB1*-related changes summarized in Table 4.

### 3.6. FGF/FGFR Pathway

*FGFR genes and their functions*. The *FGFR* genes (1–4) encode fibroblast growth receptor proteins (FGFRs; FGFR1–4), which are membrane receptors for fibroblast growth factors (FGFs). The FGF family comprises 22 proteins, which are classified into seven subfamilies, with all subfamily members being structurally related (see Table 5) [288,289,290,291,292,293,294,295,296,297]. Note that there is no FGF15 in humans.

Interestingly, most paracrine FGFs bind to FGFRs via extracellular heparan sulfate proteoglycans (HSPGs), whereas the FGF11 subfamily members act intracellularly, and the FGF19 subfamily proteins act in an endocrine manner via the αKlotho/βKlotho cofactors [289,290]. Binding to the Klotho proteins instead of the HSPG-rich extracellular matrix makes it possible for endocrine FGFs to enter the blood vessels before binding to their receptors. Moreover, several FGFs can be transferred to the nucleus via their nuclear localization signals, where some of the receptors can also be transferred. When both the FGF and FGFR are intracellularly located, they enhance gene transcription via multiple mechanisms, such as epigenetic modulations. Some activating mutations, such as *FGFR2* Y376C in EC, result in increased perinuclear protein localization in metastatic cells [289].

*The role of FGF/FGFR in signal transduction pathways.* The FGF/FGFR system is important in a broad spectrum of cellular functions, including development, metabolism, cell proliferation, apoptosis, cell migration, and angiogenesis. The single-pass transmembrane receptor FGFRs have an intracellular tyrosine kinase domain at the C-terminus [297], activating numerous downstream pathways, such as PI3K/AKT/mTOR, RAS/RAF/MEK/ERK1/2 or MAPK, PLCγ and PIP2/3/DAG/PKC, JAK-STAT, p53, and β-catenin pathways [298,299,300], with several of these pathways also interacting with each other. An exception to the downstream signaling pathway for the FGFs is FGFR-like protein 1 (FGFRL1), which lacks the intracellular tyrosine kinase domain but acts as an adhesion protein with likely no effect on cell growth and proliferation [301]. FGFRL1 deficiency, however, reduced tumor cell motility in vitro and decreased tumor growth in a xenograft model of esophageal squamous cell carcinoma [302].

FGFR signaling can be altered by other, noncanonical binding partners, such as adhesion molecules (cadherins, integrins, and the Ig superfamily of cell adhesion molecules) and extracellular matrix proteins, making this pathway even more complex. In addition, FGFRs are negatively regulated by multiple processes, including endocytosis and negative feedback phosphorylation events [289,299,303].

In tumor cells, FGFRs can be activated in both a ligand-dependent and a ligand-independent manner [299]. In the ligand-dependent manner, (1) gene amplification may result in an increased number of receptors (i.e., in breast cancer, lung cancer, or gastric cancer); (2) a gain-of-function mutation can lead to constitutive receptor activation (i.e., in breast cancer, endometrial cancer, and lung cancer); (3) FGF production is increased via the tumor microenvironment or by the tumor cell itself (also known as the “corrupted autocrine/paracrine route”); (4) gene fusion via chromosomal rearrangements leads to the creation of hybrid oncogenic FGFRs by fusing with binding partners at the carboxyl or amino termini [290].

*FGF and Klotho proteins.* Klotho proteins act as tumor suppressors in various cancers [293,304], including endometrial carcinomas, where increased βKlotho gene expression was associated with lower clinical stages of the disease [305]. In addition, *KLOTHO*, or its protein product αKlotho, has been shown to exert a tumor suppressor effect in BC and OC, among other tumors [293,294,306,307]. Their tumor suppressor effect is thought to be via the inhibition of insulin/IGF1 and β-catenin/Wnt signaling or via their effect on p53 and a subsequent reduction in cancer cell proliferation, survival, and autophagy [304].

The metabolic effects of FGFs, specifically endocrine FGFs, are numerous and include decreased gluconeogenesis, increased glycogen synthesis, increased peripheral insulin sensitivity, increased glucose metabolism, decreased lipogenesis, and increased fatty acid oxidation by FGF19. Among other effects, FGF21 has been shown to induce PGC-1α, mitochondrial energy production, hepatic gluconeogenesis, and ketogenesis [308]. In cardiac muscle cells [309] and in a cancer cell line [310], the FGFR1-like receptor was also detected in the mitochondria, where it activated pyruvate dehydrogenase (PDH) kinase 1 (PDHK1), resulting in PDH inhibition and reduced pyruvate-to-acetyl-CoA conversion and subsequently reduced glycolysis and an enhanced Wartburg effect [310]. In addition, it is likely that the FGF/FGFR signaling pathways have further significant effects on mitochondria via their downstream pathways.

Moreover, Klotho inhibits autophagy, inhibits glycolysis (via hexokinase/HK, phosphofructokinase 1/PFK1, PK M2, and PDK1), fatty acid synthesis, and purine metabolism—some specific to cancer cell effects—making it a promising drug target candidate [311,312,313]. In addition, it decreases the expression of GLUT1 and four glucose transporters, as well as the lactate transporter MCT4 [313].

*Increased FGF expression in gynecological malignancies*. Increased FGF expression via the previously mentioned “corrupted autocrine/paracrine route” has also been investigated in gynecological malignancies. In serous OCs, FGF1 overexpression was shown to correlate with tumor microvessel density and adverse outcomes [314]. A worse prognosis was also found to be associated with FGF1 single-nucleotide polymorphism (SNP) rs7727832 in OC patients [315]. Several other *FGF*/*FGFR* SNPs, however, were associated with a reduced risk of OCs, favorable treatment responses, or longer survival [315]. In addition, FGF18 overexpression was shown to be an independent prognostic marker for poor outcomes in OC patients [316], highlighting their complex role in the pathogenesis of OCs.

*FGFR* alterations have been described in many tumors, with the following tumors showing the highest percentages: urothelial cancer (32%), BC (7–23%), EC (∼13%; FGFR2 ~10–12%), squamous lung cancers (∼13–60%), and OC (∼9%) [317,318,319,320]. In type I EC, *FGFR2* alterations are generally associated with a poor outcome [199].

Moreover, *KLOTHO* gene polymorphism of the F allele of F352 V was found to be protective against BC and OC susceptibility in a meta-analysis study [321].

Targeted treatments of various tumors with *FGFR* alterations include tyrosine kinase inhibitors (TKIs), monoclonal antibodies, or ligand-binding inhibitors, and several of these have been used in clinical studies or are already approved as treatments—with TKI resistance being a concern in *FGFR*-driven tumors. The mechanism of resistance seems to be via alternatively activated signaling pathways, including PTEN upregulation and others, which are challenging to overcome [290,322,323,324]. In addition, there is a large number of FGFR inhibitors that have been or are being tested in clinical trials, with two, Erdafitinib and pemigatinib, already approved for urothelial cancer and cholangiocarcinoma, respectively [320,324]. Mitochondrial-targeted therapy, however, could improve the therapeutic effect in *FGFR*-driven tumors.

See the summary of FGF/FGFR/Klotho effects in Table 4.

### 3.7. TP53

TP53 in carcinogenesis. Tumor Protein 53 (*TP53*) was discussed in detail along with its relationship with mitochondria in our previous article (see more details in [1]). In short, *TP53* is a tumor suppressor gene coding the protein p53. It regulates a wide range of cellular functions, including cell cycle arrest, growth arrest, DNA repair, increased senescence, increased autophagy (in an mTOR-dependent and mTOR-independent manner), and reduced ROS production [1].

TP53 in mitochondria. Its mitochondrial effects are summarized as follows: *TP53* increases apoptosis (decreased Bcl2 and BclXl; increased Bax and Bak), promotes glycolysis, increases OXPHOS, inhibits the pentose-phosphate pathway, decreases glucose receptor expression, and inhibits fatty acid synthesis. In addition, it blocks the fission protein Drp1, resulting in highly interconnected mitochondria. *TP53* also enhances autophagy and reduces ROS production [1].

TP53 as a prognostic factor. In general, TP53 mutation is associated with inferior outcomes in many tumors. In EC, p53 immunohistochemistry is a significant prognostic factor, with double-positive estrogen receptor β and p53 associated with an increased incidence of metastasis and/or recurrence [325].

The frequency of *TP53* mutations in OC depends on its grade and histologic type. In high-grade serous tumors, it can be as high as 96%, and it can be as low as 8% in low-grade serous tumors. In addition, in mucinous carcinoma, the frequency is ~57%; in clear-cell OC, it is ~10%; and in endometrioid OC, it is variable (~5–55%) [247].

In addition to conventional chemotherapy, targeted treatments have been a focus of interest in treating *TP53*-mutant tumors. These include small molecules and compounds subverting the oncogenic activities of mutant p53 into wild-type p53 tumor suppressor functions [326,327]. Gene therapy, for example, with the CRISPR/Cas9 system, immunotherapy (PC1CTM, INGN-225, or H2-scDb), or increased mutant p53 degradation are all possible future therapeutic directions [328]. In addition, further possible targeted treatments of *TP53*-driven tumors include mutant p53 synthetic lethal genes [328,329]. Additional therapeutic targets, such as mitochondria-targeted therapy, are also important in these tumors, which are often chemotherapy-resistant. Targeting cellular metabolism, mitochondrial dynamics, or autophagy may be useful additional therapies to improve patient outcomes.

Please also see the *TP53* effects in Table 4.

## 4. Epithelial Ovarian Carcinomas (OCs)

*Histological subtypes, grading of OCs, and their genetic background*. Among all ovarian malignancies, epithelial OC is the most common, accounting for ~90% of ovarian tumors [330]. In 2004, the dualistic model proposed by Kurman and Shih classified OCs into type I (low grade) and II tumors (high grade) according to their epithelial pathogenesis [331]. Type I tumors often have precursor lesions and include endometrioid, mucinous, and clear-cell carcinomas, whereas type II tumors include high-grade serous carcinoma and carcinosarcoma. In type I tumors, the common mutations found are *BRAF*, *KRAS*, and *PTEN*, with *KRAS* and *BRAF* being mutually exclusive [332]. Of the mutations seen in type I carcinomas, somatic mutations in *PTEN* are more common in endometrioid-type OC. On the other hand, type II tumors are frequently associated with alterations in *TP53*, *BRCA1*, and *BRCA2*.

High-grade serous OC accounts for ~70–80% of OCs, typically presenting at a late stage and often with disseminated disease [333]. *TP53* alterations have been found in almost 100% of high-grade serous OCs. Other commonly associated gene alterations include *BRCA1*, *BRCA2*, *NF1*, *CDK12*, Homologous Recombination Repair genes, and alterations in the PI3/Ras/Notch/FoxM1 pathway.

Low-grade serous OC accounts for ~10% of serous OCs. Although they are more indolent compared to high-grade serous ovarian cancer, they are commonly diagnosed at an advanced stage with poor overall survival due to an inadequate response to chemotherapy and hormonal agents [333,334]. The associated genetic alterations include *KRAS*, *NRAS*, *BRAF*, *ERBB2*, and *PI3KCA* oncogenes [334].

Endometrioid OC accounts for ~10% of epithelial OCs [335]. Genomic analysis has identified pathogenic somatic variants in *ARID1A*, *PIK3CA*, *PTEN*, *CTNNB1*, and *PP2R1A*. Microsatellite instability has also been found to result from mismatch repair deficiency [333,334]. It is important to note that the predictive value of PTEN inactivation is uncertain but has been associated with chemotherapy resistance [336].

Clear-cell OC accounts for ~5% to 10% of epithelial OCs in the post-menopausal population [335], with the most common genetic alterations including *ARID1A*, *PIK3CA*, *PTEN*, *CTNNB1*, and *PP2R1A* genes [334]. Of the above gene alterations, *ARID1A* variants occur in ~50% of clear-cell cases, and *PIK3CA* variants occur in approximately ~36% [333]. Clear-cell and low-grade endometrioid epithelial OCs associated with endometriosis frequently harbor *ARID1A* mutations [337]. Additionally, *PIK3CA* mutation is a possible early event in the transformation of endometriosis into clear-cell OC [338]. The frequently seen PI3K/AKT/mTOR pathway activation in OC is associated with a poor survival rate and also with more invasive disease, with the tumor cells having better migratory abilities [339,340,341]. Interestingly, PI3K/AKT/mTOR pathway changes may co-exist and collaborate with other genes in tumorigenesis. In one study, *PIK3CA* mutations were detected in 40% (17/42) of clear-cell OCs, with 71% of these cases also having lost *ARID1A* expression [342]. Furthermore, p53 may suppress *PIK3CA* transcription through direct interaction with its promoter in ovarian surface epithelial cells [343,344].

In addition, FGFR alterations are seen in ∼9% of OCs, whereas *TERT* promoter mutations are seen in ~16% of clear-cell OCs [317,345].

The treatment of epithelial OCs is still limited, with often a poor response to conventional chemotherapy, immunotherapy, and surgical resection [346,347,348]. New therapeutic targets are therefore essential in order to improve patient outcomes in this disease.

Here, we further discuss mitochondrial changes in epithelial OCs. In addition, we discuss *ARID1A* and *TERT* mutations and their relation to mitochondrial changes. Note that many genes important in the pathogenesis of OCs have already been discussed in previous sections. Additionally, see Table 4 for a summary of the effects of these genes.

### 4.1. OC and Mitochondria

Mitochondrial changes, as in other types of tumors, are important in the development of OC and in the survival of tumor cells. Although certain gene mutations could also help to identify potential mitochondria-related therapeutic targets in OC (which are discussed separately), there are mitochondrial changes associated with the disease in general.

*Mitochondrial mass*. Increased mitochondrial mass with enhanced mtDNA transcription/translation via increased PGC1α and TFAM and an increased mtDNA copy number have been described in OC cells [7,349,350]. The mtDNA copy number, however, decreases with cancer progression. Interestingly, the ovarian mtDNA copy number also decreases with age [350].

*Circulating free mtDNA*. The circulating cell-free plasma mtDNA level was found to be increased in epithelial OCs when compared to benign ovarian diseases. However, in this study, it increased with cancer progression but decreased after chemotherapy [351,352,353]. Additionally, in a small serous OC study (n = 24 cancer patients), the exosome-encapsulated mtDNA copy number was significantly increased in all stages of the disease, whereas the mtDNA copy number from circulating cell-free plasma was not significantly changed compared to healthy controls. Interestingly, the whole-blood mtDNA copy number showed opposite results: it was significantly decreased in cancer patients of all FIGO stages compared to controls [351].

*mtDNA mutations in OCs*. As for mtDNA mutations, mutations in both the D-loop and coding regions have been associated with OC risk and/or disease. In addition, nuclear DNA-coded mitochondrial biogenesis genes and other mitochondrial protein-coding genes have also been found to be altered in OC, many contributing to chemotherapy resistance. For a detailed review see [7].

*Changes in mitochondrial metabolism in OCs.* Metabolic changes are vital in the pathogenesis, progression, and therapy resistance of epithelial OCs [250], providing a survival benefit to the tumor cells, with mitochondrial changes being at the center of these alterations. When investigating the glycolytic activity of OC cell lines compared to normal ovarian cells, most studies showed increased activity, with only a few cell lines showing the opposite results. Moreover, OXPHOS seems to be dominant in most OC cell lines and even more pronounced in invasive OC cell lines [354,355,356,357]. Some other studies, however, showed decreased OXPHOS in OC [354]. Anaerobic glycolysis is increased in OC tumor tissue, especially in high-grade serous OC and clear-cell OC subtypes. Importantly, inhibiting the anaerobic glycolytic pathway in in vitro and in vivo OC models could effectively induce tumor cell death and reduce tumor cell growth, invasion, migration, and angiogenesis. An interesting in vitro study that used chemotherapy-resistant OC cell lines found that the individual cells had different glucose metabolism profiles: (1) anaerobic glycolysis-predominant; (2) OXPHOS-predominant; (3) showing both strong glycolytic and OXPHOS activities. Importantly, blocking the dominant glucose metabolism pathway in these cells could enhance cytotoxicity and/or chemosensitivity [354]. These results may also explain why other studies showed different OXPHOS activities in vitro in OC cells. For more details on glucose metabolism studies in OC, see [354]. Additionally, increased GLUT1 expression was found in various OC models and tissues, which contributed to enhanced tumor proliferation. Additionally, higher expression was seen in higher stages of the disease—associated with shorter disease-free survival [8,250,358,359]. Moreover, in in vitro and in patient-derived xenograft OC models, blocking GLUT1 with BAY-876 resulted in glycolysis and tumor growth inhibition [8]. A study using semiquantitative immunohistochemical staining of paraffin-embedded OC tissue found that the overexpression of sodium-glucose co-transporter-1 (SGLT1), an active membrane glucose transporter, was a poor independent prognostic marker [250,360].

Abnormal fatty acid synthesis and its enhanced metabolism are also hallmarks of OC cells, with higher fatty acid synthase expression correlated with decreased survival rates [250,361]. This is likely due to the fact that tumor cells that are more proliferative have increased ATP needs that cannot be supported by only using glucose. These cells therefore are more resistant to environmental changes, including relative glucose deprivation. Increased fatty acid metabolism is also linked to cisplatin resistance in OC [250,362]. Amino acid metabolism pathways, such as histidine, tryptophan, arginine, proline, alanine, aspartate, and glutamine metabolism pathways, were shown to be involved in OC tumor growth in a clinical study [363]. These data support the possible use of fatty acid and/or amino acid metabolism inhibition in OC therapy.

*Changes in mitochondrial dynamics in OCs*. Mitochondrial dynamics is also altered in OC. Previous data show an increased mitochondrial fission tendency and/or increased Drp1 fission protein or mRNA expression in various OC models [355]. In addition, analyzing TCGA data showed that OCs had one of the highest *DNML1* gene amplification events amongst all the cancers represented—although only present in ~8% of samples. Furthermore, ~11% of high-grade serous OCs showed increased Drp1 mRNA expression [355]. The increased expression of the mitochondrial fission protein (Drp1)-encoding gene, *DNML1*, has been linked to poorer survival and chemoresistance in OC [364]. In line with this, mitochondrial fission inhibition targeting Drp1 results in chemosensitivity in vitro [365]. On the other hand, a 2014 in vitro study by Kong and co-workers showed that chemoresistant OC cells had more fused mitochondria than chemosensitive cells [366]. Additionally, nutrient starvation in addition to Bcl-2 mimetic treatment resulted in excessive mitochondrial fission in an in vitro OC study [367,368]. Importantly, Drp1 inhibition is a promising therapy in OC, potentially for both fission- and fusion-predominant OC cells. Increased fission in fusion-predominant cells disrupts their metabolism and may induce apoptosis. However, pushing fission to the extreme also results in cell death in many models via dysfunctional mitochondrial fragment production, subsequently increasing ROS production [369,370].

### 4.2. ARID1A

*The role of ARID1A in carcinogenesis*. ARID1A (AT-Rich Interaction Domain 1A) is a bona fide tumor suppressor gene, given its loss of function in many different tumors. Under specific circumstances, however, it acts as a proto-oncogene (more on this later in this section) [371].

The *ARID1A* gene encodes the ARID1A protein, which is a line-restricted (cell-line-specific) member of the BRG1-associated factor (BAF) complex—the human equivalent of the SWItch Sucrose Non-Fermentable (SWI/SNF) chromatin-remodeling complex, which is responsible for ATP-dependent chromatin remodeling, ubiquitously expressed in all cell types [2,372,373,374,375]. ATP-dependent chromatin remodeling is one of the epigenetic mechanisms by which DNA expression and chromatin accessibility are modulated, along with DNA methylation and histone modifications [1,373,376]. In addition to the BAF complex, mammals have other ATP-dependent chromatin remodeling units, namely, INO80/SWR1, ISWI, and CHD [373]. Mammalian BAF complexes are large protein complexes containing up to 15 subunits, with variable subunit combinations existing depending on the tissue type and stage of cell maturation. In most tissues, three main subtypes of BAF complexes can be found: the canonical BAF (simply BAF or cBAF), the polybromo-associated BAF complex (PBAF), and the noncanonical GLTSCR1/1L-BAF complex (GBAF/ncBAF). The possible subunit constitutions, however, result in over a thousand possibilities for BAF and PBF complexes [377]. The presence of the correct specific subunits is therefore important both during development and for appropriate tissue-specific functions [373].

The BAF complex has to include one of two mutually exclusive catalytic ATP-ase subunits, both showing helicase activities: SMARCA2/BRM or SMARCA4/BRG1; the latter is found, for example, in embryonic stem cells. In addition to these, there is a set of widely expressed core units (SMARCB1/SNF5/INI1; SMARCC1/BAF155; and SMARCC2/BAF170) and several different line-restricted subunits (such as ARID1A/BAF250A; ARID1B/BAF250B; SMARCD1,2,3/BAF60A,B,C; SMARCE1/BAF57; etc.) that make up the specific BAF complexes [373,374,378]. Importantly, AIRD1B is only found in the BAF/cBAF complex. See Table 6 for a summary of BAF subunits (please note that most subunits have alternative names) [378,379].

Different subunits are associated with different tumors: for example, SMARC1 loss is seen in atypical teratoid/rhabdoid tumors (ATRTs), among others [380,381], whereas AIRD1A loss has been described in endometrioid EC (~40%), endometrioid (~30%), clear-cell (46–57%) OCs, etc., with up to ~20% of all human cancers harboring *AIRD1A*-inactivating mutations [337,372,382,383,384,385]. In addition, the loss of ARID1A has been demonstrated in patient samples with atypical endometriotic lesions contiguous to clear-cell OC, raising the possibility that the loss of ARID1A may contribute to an early cancer-promoting event in endometriosis that leads to clear-cell OC [337]. Additionally, in ~13% of BCs, there is mostly a loss of heterozygosity of *ARID1A*, resulting in reduced protein expression and a more aggressive cancer phenotype [386]. In various tumors, ARID1A loss is mainly due to *ARID1A* promoter hypermethylation, but it also can be due to inactivating mutations or in-frame insertions/deletions [382,386]. In some cases, post-translational changes lead to the loss of the ARID1A protein. Importantly, ARID1A inactivation is associated with poor outcomes and reduced chemosensitivity in many tumor types, including clear-cell OCs [2,377,383,387,388,389].

BAF complexes, upon nucleosome engagement, undergo conformational changes, resulting in bilateral nucleosome interactions—both at the ATP-ase end and at the other end, such as via the SMARCB1 complex. This unique DNA binding makes these complexes very efficient in DNA remodeling, and specific to these complexes, they may even eject nucleosomes [379]. ARID domains such as ARID1A are DNA-binding domains, with other subunits typically interacting with histones and proteins. In addition, as previously mentioned, ATP-ase subunits are always present in BAF complexes [379]. Upon ATP-ase activation, the BAF complex relaxes the heterochromatin to euchromatin, making the DNA more accessible [382]. Additionally, its histone modification effect results in more or less decondensed DNA, leading to increased or decreased transcription, respectively [374]. Interestingly, certain subunits may act as transcription factors or coactivators/repressors [374]. In addition to these, BAF complexes are also involved in DNA replication, methylation, and damage repair [374]. Moreover, to make their cellular functions even more complex, certain subunits, such as ARID1A, also have a direct effect on other proteins, such as p53 and Rb [387,390].

*BAF complexes and their influences on other signal transduction pathways.* Because of its large scale of regulatory effects, BAF complexes alter various downstream cellular pathways, including the sonic hedgehog (SHH) pathway, the Wnt/β-catenin pathway, the E2F/CCND1/Rb pathway, the EZH2/polycomb complex pathway, and the ROCR1/RHOA pathway, and they can also alter nuclear receptor signaling (such as hormone receptors) and can directly interact with proteins, such as p53 [374,387]. Each subunit alteration, however, has been associated with specific downstream pathways—although a lot of them understandably overlap [374].

ARID1A per se is involved in regulating various cellular functions, such as cellular differentiation, the cell cycle, cell migration, DNA repair, angiogenesis, and cellular metabolism [2,377,383,388]. Its tumor suppressor function is more well researched, with *ARID1A* inactivation resulting in tumor initiation in *PTEN*- or *PIK3CA*-mutant cells in gynecological malignancies and hypermutated/MSI-type cells in colon cancer [383]. In some preclinical studies, however, *AIRD1A* inactivation was insufficient to be the sole driver mutation for tumorigenesis, with rare studies linking *ARID1A* loss to slower tumor initiation [371,383,390].

*ARID1A and BAF complexes in cellular metabolism and mitochondria*. The effects of the BAF complex and ARID1A on cellular metabolism and mitochondria are numerous. In a recent in vitro OC study, Zhang and co-workers showed that ARID1A loss resulted in increased mitochondrial membrane potential, increased mitochondrial mass, and enhanced mitochondrial fission, likely at least in part due to increased c-Myc expression [391].

*ARID1A-* and *SMARCA4*-mutant tumors were found to be more sensitive to the inhibition of OXPHOS when compared with wild-type controls in a lung cancer study [2,392]. In addition, the inhibition of mitochondrial complex I, using IACS-010759, showed an extended overall survival in a preclinical model of *ARID1A*-mutated clear-cell OC [391]. In addition to increased OXPHOS, enhanced anaerobic glycolysis (with increased Pgam1, pyruvate kinase M, and Pgk1 expression) has also been described in a lung cancer model with ARID1A loss, where a small-molecule bromodomain and extraterminal protein (BET) inhibitor (JQ1) was successful in treating tumor cells via glycolysis inhibition [393].

Furthermore, ARIDA1 affects several downstream pathways, such as [382,390,394,395,396]:

1. EZH2, histone modifications (HDACs);
2. SHH (ARID1A loss, possibly resulting in inhibition);
3. p16 and p21, CDK4/5, Rb, and E2F4 (ARID1A loss, resulting in inhibition);
4. Wnt/β-catenin (ARID1A loss, resulting in activation);
5. *TP53*, p53 (ARID1A loss, resulting in inhibition);
6. TERT (ARID1A loss, resulting in activation);
7. Transforming growth factor β (TGFβ) (ARID1A loss, resulting in inhibition);
8. MYC (ARID1A loss, resulting in activation);
9. KRAS (ARID1A loss, resulting in activation);
10. PI3K/AKT/mTOR (ARID1A loss, resulting in activation).

In addition to these, functional ARID1A has an important role in DNA damage repair [394]. It also regulates nuclear hormone receptors, such as the ER, which is upregulated when ARID1A function is intact [390]. Furthermore, ARID1A loss has been linked to replication stress, uncontrolled DNA replication, Ang-2-dependent angiogenesis, enhanced cell migration, and invasion [382,390]. Moreover, ARID1A loss results in YAP upregulation (YAP/TAZ pathway) and a resultant enhancement of cell proliferation [390]. Furthermore, ARID1A deficiency results in increased IL-6 expression and JAK/STAT activation [397] and has an increased response to immune checkpoint inhibitors [390]. There is no information about ARID1A per se, but the loss of the BAF complex or SMARCB1 alters ROCK1/RHOA expression, leading to increased cell motility [374,398].

**Figure 4:**
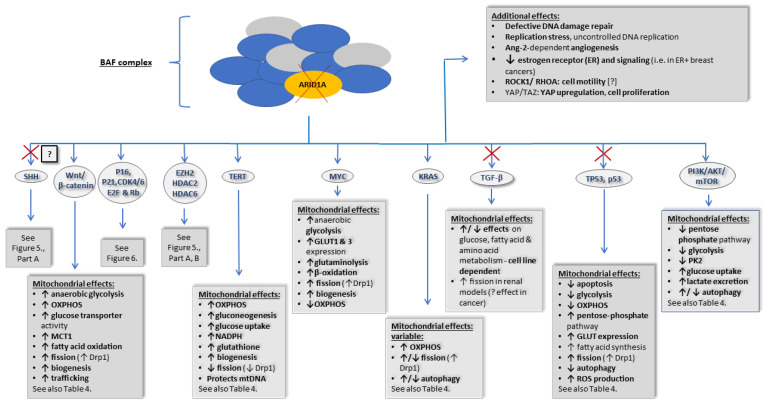
The effects of ARID1A loss on cell function and on its downstream pathways. *Abbreviations: ARID1A*: AT-Rich Interaction Domain 1A; *BAF*: BRG1-associated factors; *CDK*: cyclin-dependent kinase; *EZH2*: enhancer of zeste homolog 2; *SHH*: sonic hedgehog; *TERT*: telomerase reverse transcriptase; *TGF*: transforming growth factor; *Wnt*: Wingless/int1. Blue arrows: activation or showing effect/result; red lines: inactivation/loss/blocking.

Altering various mitochondrial functions therefore could be beneficial in tumors with ARID1A loss (also see below for the effect of the other downstream pathways on mitochondria). See Figure 4 and subsequent sections for further details.

#### 4.2.1. EZH2 and HDACs

Epigenetic modulations, such as DNA methylation or histone modifications, are discussed in detail in our previous article (Czegle and co-workers, 2021) [1].

EZH2. In short, enhancer of zeste homolog 2 (EZH2) is an epigenetic modulator and is part of the polycomb repressive complex 2 (PRC2). PRC2 is a key gene transcription inhibitor acting via the trimethylation of lysine 27 of histone H3 (H3K27). EZH2 can be induced at the transcriptional level or by post-transcriptional modifications. When EZH is inhibited or knocked down, reduced glycolysis, enhanced OXPHOS, and reduced fatty acid synthesis are seen in vitro, along with apoptosis inhibition. Its effect on mitochondrial dynamics, however, has not been elucidated [1,373].

With the loss of ARID1A, inhibition via EZH2 is lost, subsequently increasing H2K27 methylation and making the transcription sites less accessible [382]. In preclinical studies, the proliferation of ARID1A-mutated cells was successfully inhibited [384,390]. Given EZH2’s effect on mitochondria and metabolism, its inhibitors likely act at least in part by altering cellular metabolism in EZH2-induced tumors, which is typically associated with drug resistance.

Histone deacetylases (HDACs). Histone deacetylases (HDACs) remove acetyl groups from histones, resulting in a more closed chromatin structure and thus interfering with gene transcription. HDAC enzymes were found to be overexpressed in various malignancies, such as those with ARID1A loss [1,382,390]. As per previous studies, ARID1A loss induces the transcription of *HDAC6*, along with *AURKA* and *TERT* [382]. HDAC6 has a significant effect on protein trafficking, cell migration, and cell cycle regulation (i.e., via p16 [399]). In addition, HDAC6 inhibition largely inhibited tumor growth in *ARID1A*-mutant tumors. Moreover, HDAC6 inhibition improved animal survival in a clear-cell OC model when given with an anti-PD-L1 immune checkpoint inhibitor [390,400]. In addition to HDAC6, ARID1A loss also affects HDAC1, likely at least in part by direct protein–protein interaction, with an expected loss of binding between the two proteins with ARID1A loss [377,401]. HDAC2 activity is also increased with ARID1A loss [402].

HDAC1/2 inhibition resulted in c-Myc-dependent glycolysis inhibition, enhanced OXPHOS, fatty acid oxidation, and mitochondrial biogenesis in a GBM model [403]. In addition, HDAC6 activation increases mitophagy [404].

**Figure 5:**
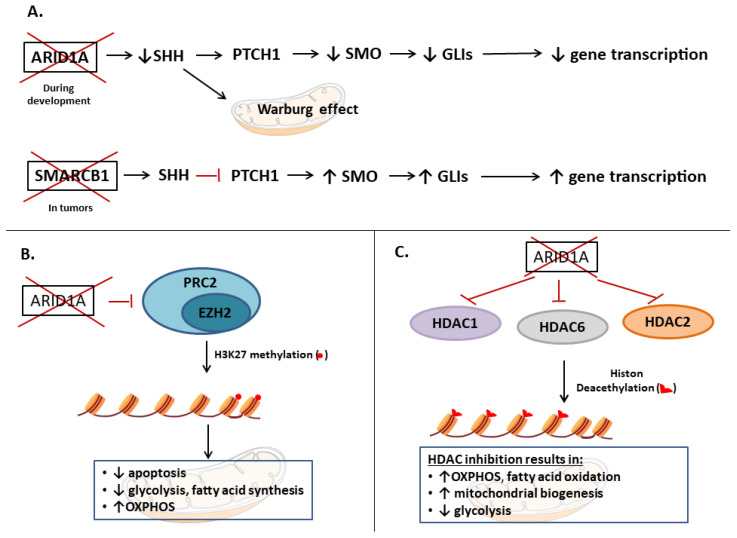
The effects of ARID1A loss on the SHH pathway (**A**), on EZH2 (**B**), and on HDACs (**C**). A: ARID1A loss has been linked to metabolic changes, resulting in the Warburg effect via alterations in the SHH pathway during development, with no data existing on cancer cells. In addition, ARID1A loss was shown to decrease SMO and GLI expression. SMARCB1 loss, on the other hand, shows SHH pathway activation and increased GLI1 and PTCH1 expression in tumor cells. B: EZH2, which is part of the PRC2 complex, inhibits transcription via histone H3K27 trimethylation, which results in more condensed DNA. With ARID1A loss, there is increased H3K27 methylation. C: Histone deacetylation via HDAC also results in more condensed DNA. With ARID1A loss, there is increased histone deacetylation. Abbreviations: ARID1A: AT-Rich Interaction Domain 1A; EZH2: enhancer of zeste homolog 2; GLI: glioma-associated oncogene homolog; HDAC: histone deacetylases; OXPHOS: oxidative phosphorylation; PRC2: polycomb repressive complex 2; PTCH1: Patched1; SHH: sonic hedgehog; SMO: Smoothened. Black arrows: activation/results in; red lines: inhibition/deletion/loss; ↑: increase; ↓: decrease.

See also Figure 5B,C.

#### 4.2.2. SHH Pathway

The sonic hedgehog (SHH) pathway functions to regulate cell homeostasis, differentiation, proliferation, and vasculogenesis. The SHH protein is part of the hedgehog protein family, comprising two other ligands (Indian hedgehog/IHH and Desert hedgehog/DHH) besides SHH, named after their activating ligands [398,405,406,407,408].

In the canonical pathway, the SHH protein binds to one of the cell membrane receptors, Patched 1 (PTCH1). After SHH binding, PTCH1 becomes inactivated and can no longer inhibit the SMO (Smoothened) protein, which then translocates to the plasma membrane from the cytoplasm and frees the glioma-associated oncogene homolog (GLI) zinc finger transcription factors (GLI1, GLI2, and GLI3). GLIs subsequently translocate to the nucleus, where they activate their target genes. In the absence of the ligand, PTCH1 inhibits SMO, which then remains in the cytoplasm, where it eventually degrades. As a result of this, GLI target genes are not activated [398,405,406,407,408]. When GLI transcription factors are activated, they can induce tumorigenesis, tumor growth, and chemoresistance [405].

The noncanonical SHH pathway, on the other hand, can be classified into three types: (1) PTCG-mediated, (2) SMO-dependent/GLI-independent, and (3) SMO-independent GLI activation. These, however, include any SHH effect that is not the canonical pathway, including downstream metabolic effects and mitochondrial changes.

*HH pathway and cellular metabolism*. The activation of the HH pathway has a significant effect on glucose metabolism. Mouse cell line studies showed that both active and inhibited SHH/SMO signaling can induce the “Warburg effect”, with the latter occurring via the AMPK pathway [409]. In addition, SHH pathway inhibition resulted in increased cellular glucose uptake in an in vivo mouse study [409]. On the other hand, the partial agonism of the HH pathway also resulted in a “Warburg-like” effect in another study [410]. In adult mice, the activation of SHH signaling enhances insulin responsiveness and fatty acid oxidation. At the cellular level, SHH did not affect glucose uptake but increased GLUT-4 and insulin receptor KLB1 expression in astrocytes [411].

Furthermore, active SHH signaling was shown to decrease mitochondrial fission via Drp1 inhibition in an endometrial hyperplasia model [412].

During neuronal development, the npBAF complex suppresses the SHH pathway [413]. In addition, during dental root progenitor proliferation and differentiation, ARID1A loss impaired differentiation and resulted in cell cycle arrest via HH signaling regulation [394]. These effects, however, might be different in tumorigenesis, where ARID1A loss would be expected to enhance proliferation. Of note, SMARCB1-deficient tumors show SHH pathway activation and increased GLI1 and PTCH1 expression [381]. Therefore, the effect of ARID1A loss on tumorigenesis is yet to be determined.

The effects of ARID1A loss on the SHH pathway are summarized in Figure 5, part A.

#### 4.2.3. p16 and p21, CDK4/5, Rb, and E2F4

The p16 protein (also known as p16^INK4a^), encoded by the cyclin-dependent kinase inhibitor 2A (*CDKN2A*) gene, is a tumor suppressor, inhibiting cyclin-dependent kinases (CDK4/6) that maintain Rb in a phosphorylated state when bound to cyclin D1 (encoded by *CCND1*) [381,414,415]. The phosphorylated, functionally inactive Rb protein can subsequently liberate the E2F transcription factor, resulting in the expression of several proliferation-related genes essential for G0/G1 progression [375,381,398,414,415]. Although the proapoptotic function of Rb is mostly linked to BAX-dependent mitochondrial permeabilization [416], recent evidence supports that p16 itself also regulates mitochondrial biogenesis and mitochondrial function independently of its action on this canonical CDK/Rb pathway [376,414,417].

These changes were studied in an in vitro model, which showed that p16-deficient cells have a higher mitochondrial mass with the increased expression of the mitochondrial transcription of PGC-1-related coactivator (PRC) and transcription factor A (TFAM), the increased expression of mitochondrial respiratory subunit proteins, and, paradoxically, decreased respiratory capacity. These subsequently resulted in enhanced mitochondrial ROS generation, which can promote cellular migration [376]. Moreover, p16 overexpression resulted in the decreased expression of certain mitochondrial respiratory proteins, increased respiration, and decreased migration in an in vitro melanoma model [376]. These functions were all independent of the CDK4/Rb pathway and cell cycle control [376].

A previous study showed that *ARID1A* knockdown leads to decreased *CDKN2A* expression with subsequently increased cell proliferation in a *KRAS*-mutant pancreatic cancer cell line [418]. In addition, ARID1A loss increased cyclin D1 expression in another pancreatic tumor cell line experiment [419].

Additionally, cyclin D-CDK4 expression is associated with increased mitochondrial biogenesis, whereas CDK4 inhibition has the opposite effect in Drosophila [420]. Among others, *CCND1* transcription inhibition can be due to the direct recruitment of HDACs to the *CCND1* promoter region by a functional BAF complex/*SMARCB1* and likely *ARID1A* [375,381,398,414,415]. Reduced *CCND1* transcription then results in decreased cyclin D1 expression, resulting in cellular senescence. On the contrary, BAF/SMARCB1 inactivation results in increased cell proliferation [375,381,398,414,415].

Furthermore, ARID1A loss reportedly increases p21 expression, resulting in increased angiogenesis. Besides its effect on angiogenesis, p21 is also important in cell cycle progression and has been reported to increase the mitochondrial mass (without increased OXPHOS) [390].

**Figure 6:**
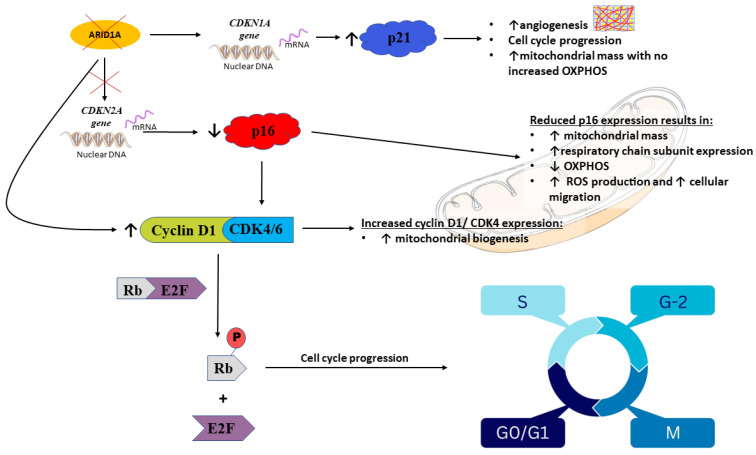
ARID1A loss and its effects on p16, p21, and cyclin D1. ARID1A loss results in increased p21 expression and subsequently enhanced angiogenesis, increased mitochondrial mass, and cell cycle progression. Furthermore, ARID1A loss results in decreased p16 expression. This decreased p16 expression and ARID1A loss itself then subsequently increase cyclin D1/CDK4/6 expression and binding, which in turn maintains Rb in a phosphorylated state. Phosphorylated Rb subsequently liberates the E2F transcription factor, resulting in cell cycle progression. Increased cyclin D1/CDK4/6 also results in increased mitochondrial biogenesis. Moreover, decreased p16 expression has also been associated with increased mitochondrial mass, increased OXPHOS and respiratory chain protein expression, increased ROS production, and enhanced cell migration. Abbreviations: ARID1A: AT-Rich Interaction Domain 1A; CDK: cyclin-dependent kinase; CDKN: cyclin-dependent kinase inhibitor; OXPHOS: oxidative phosphorylation; Rb: Retinoblastoma protein; ROS: reactive oxygen species. Black arrows: activation/results in; red lines: inhibition/deletion/loss; ↑: increase; ↓: decrease.

The effects of ARID1A loss on p16, p21, and cyclin D1 are summarized in Figure 6.

#### 4.2.4. Wnt/β-Catenin Pathway, TP53/p53, TERT, and KRAS

ARID1A loss results in the activation of the Wnt/β-catenin pathway [421]. Its effects on mitochondria are discussed in Section 3.5.

ARID1A enhances *TP53* expression and also directly binds to the p53 protein. ARID1A loss therefore results in inhibited *TP53* expression/p53 effect [387,422]. For more details on *TP53*, see Section 3.7.

ARID1A negatively regulates *TERT* transcription by directly binding to its promoter region and via histone modification [382,395]. For more details on *TERT*, see Section 4.3.

ARID1A loss results in the activation of the RAS pathway [419]. For more details on the pathway itself, see Section 3.4.

See these effects also in Figure 4.

#### 4.2.5. Transforming Growth Factor β (TGF-β)

ARID1A loss results in TGF-β inhibition, leading to increased cell proliferation [382,396]. TGF-β is a cytokine that, upon binding to one of its cell membrane receptors, TGF-β receptor type I or II, leads to intracellular SMAD signaling activation. TGF-β typically acts as a tumor suppressor in normal tissues and in the early stages of tumorigenesis, but it is an oncogene during the later stages of cancer maintenance and progression [423]. The effects of TGF-β on metabolism are diverse, with often opposite effects in different cell types and in normal versus cancer cells. These include increased or decreased OXPHOS, anaerobic glycolysis, glucose uptake, and lipid and amino acid metabolism, with no data on OC or EC. In BC, increased OXPHOS, glutaminolysis, and fatty acid synthesis and oxidation were described [423,424]. For a detailed review, see [424]. In addition, TGF-β induces mitochondrial fission in renal fibrosis [425,426], with no data on tumorigenesis. See these effects also in Figure 4.

#### 4.2.6. MYC

ARID1A loss results in *MYC* activation [371], with the role and effect of *MYC* discussed in detail in our previous manuscript [1]. In short, c-MYC/MYC, encoded by *MYC*, is a transcription factor that alters the expression of a large number of genes. Similarly to *RAS*, *MYC* activates *TP53*, enhances ROS production, and causes replication stress. Furthermore, *MYC* increases glycolysis, the expression of glucose transporters (GLUT1 and GLUT3), lactate transporters, and glutamine transporters but suppresses OXPHOS. In addition, MYC increases β-oxidation and glutaminolysis. It also promotes Drp1-dependent fission and increases mitochondrial biogenesis (via increased PLOG, PLOG2, and NRF1 expression). Mitochondrial trafficking is also induced by *MYC* via RHOT1, RHOT2, TRAK2, and Kif5B [1]. See these effects also in Figure 4.

#### 4.2.7. PI3K/AKT/mTOR

ARID1A loss results in the activation of the PI3K/AKT/mTOR pathway [390]. For more details on the pathway itself, see Section 3.3. See these effects also in Figure 4.

### 4.3. TERT

Telomerase is a ribonucleoprotein composed of a catalytic component, telomerase reverse transcriptase (TERT, encoded by the *TERT* gene), and a telomerase RNA component (TERC) that is known for its function in telomere elongation [427]. In normal conditions, telomerase expression is limited to embryonic and other stem cell-like cells and is silenced in somatic cells [428], whereas *TERT* gene and *TERT* promoter alterations (discussed in more detail in articles such as [373]) are frequently detected in various cancers, resulting in telomere length protection, implicated as a key factor in tumor cell propagation and tumorigenesis [429].

*TERT functions*. In addition to its telomere elongation effect, TERT also has so-called noncanonical functions. These include the regulation of chromatin structure, the protection of mtDNA, RNA silencing, epigenetic changes, the subsequent activation of signaling pathways (such as nuclear factor κ-light-chain-enhancer of activated B cells (NF-κB) or Wnt/β-catenin signaling pathways), mitochondrial and metabolic regulation, and an increase in cell adhesion and migration [428].

*TERT in mitochondria*. Although the relationship between mitochondria and TERT is not fully understood, several interactions have been documented, including apoptosis regulation, the protection of mtDNA from oxidative stress, the regulation of glucose metabolism and uptake, mitochondrial dynamics, and autophagy. TERT, although primarily localized in the nucleus, can be shuttled to the mitochondria, but it may also reside in the cytoplasm [428]. Intramitochondrial TERT has been shown to either promote or inhibit oxidative-stress-induced apoptosis [430] following its translocation via its N-terminal mitochondrial leader sequence [431]. One study found that TERT’s effect on cell death depended on the cell stage when it underwent apoptosis. In the early stage of apoptosis, cells with high levels of mitochondrial TERT were more likely to die than those with lower levels. Conversely, in the later stage of apoptosis, higher concentrations of mitochondrial TERT correlated with a longer time to complete apoptosis [430]. Additionally, mitochondria, the main source of ROS production in the cell, can shorten telomeres due to their susceptibility to oxidative damage. Interestingly, shortened telomeres also affect mitochondria through multiple signaling pathways [427]. In one pathway, telomere dysfunction activates p53, which binds and suppresses the PGC-1α and PGC-1β promoters, leading to decreased mitochondrial biogenesis [432]. Additionally, one study demonstrated that TERT can protect mtDNA from oxidative damage by directly binding to it and by reducing ROS levels [433].

**Table 4:** The effects of EC and OC driver mutations in general and their effects on mitochondria.

Gene/Protein Name Gene Effect	General Effects ± Main Downstream Pathways	Effect on Mitochondria
*PTEN* /PTEN Tumor suppressor	PI3K/AKT/mTOR (activated via *PTEN* loss) and non-enzymatic roles *PTEN* loss results in: ↑ cell proliferation ↑ cell growth ↑ cell survival, migration, cell adhesion ↑ angiogenesis	*PTEN*: - ↑ apoptosis induction [226] - ↑ ROS production [226] Contradictory data on autophagy: - ↑ autophagy and lysosomal mass [227,228,229,230] or - ┤mitophagy via blocking the TLR4–JNK–BNIP3 pathway [231,232] -┤mitophagy via ubiquitin dephosphorylation [231,232] - ┤mitophagy via ↑ Mfn2 and ↓Rab7a [231,232] Loss of *PTEN*: - ↑ glycolysis [233] - ┤ gluconeogenesis [233] - ↑ lipogenesis [233] - ↑ mitochondrial biogenesis [233]
*PIK3CA* /PI3K Proto-oncogene	PI3K-AKT-mTOR pathway PIK3CA activation results in: ↑ cell growth ↑ motility ↑ survival and proliferation ↑ protein synthesis ↑ intracellular trafficking ↑ angiogenesis	PI3K/AKT/mTOR pathway: ┤ pentose phosphate pathway (via G6PD stabilization) [250] ┤PK2, a rate-limiting enzyme of glycolysis [250] ↑ glucose uptake [250] ↑ lactate excretion [250] ┤autophagy (at moderate ROS levels) [254] ↑ autophagy (at moderate ROS levels) [254] ┤PI3K-AKT-mTOR pathway: - ↓ intracellular lipid accumulation via ↓ de novo fatty acid synthesis [251], ↓ FASN [252], ↓ SREBP [252], and ↑ fatty acid oxidation [251] Contradictory data on mitochondrial trafficking: - PI3K and mTOR inhibitors: ┤ tubulin polymerization, leading to microtubule disturbance [256] or - PI3K inhibitor: ↑ mitochondrial trafficking [257]
*KRAS*/KRAS Proto-oncogene	Major downstream pathways: PI3K, MAPK, and Ral small GTPase KRAS activation results in: - ↑ proliferation, transformation - Cell survival	- ↑ mitochondrial fission (↑ Drp1) [1,262,263,264] - ↑ mitophagy [1,262,263,264] - ↑ OXPHOS [1,262,263,264] The effect of tumor-suppressive therapy in RAS-driven tumors: - ┤Drp1 [262,263,264] - ↑ Mfn2 expression (mitochondrial fusion induced by doxycycline/leflunomide) [263] - ↓ autophagy proteins [265]
*CTNNB1* /β-catenin Proto-oncogene	Major downstream pathways: β-Catenin regulates the expression of many Wnt target genes, including *MYC*, *CCND1*, and *CDKN1A* General effects: - ↑ proliferation - regulation of cellular development and differentiation - ↑ angiogenesis - regulation of migration and invasion - regulation of cellular homeostasis	Wnt/β-catenin activation: - ↑ anaerobic glycolysis (↑ PDK1, ↑LDH-A) [272,273,274,275,276,277,278] - ↑ OXPHOS (typically less increment than anaerobic glycolysis) [275,276,277,278,279] - ↑ glucose transporter activity [277] - ↑ MCT1 [272,278] - ↑ fatty acid oxidation [280] - ↑ mitochondrial fission (↑ Drp1) [282] - ↑ apoptosis (although under special circumstances, the opposite is true) [283] Wnt/β-catenin signaling inhibition: ↓ anaerobic glycolysis (↓ PDK1) [273] ↓ SREBP-1c in hepatocytes [281]
*FGFRs (1–4)* /FGFRs (1–4) Proto-oncogenes	Major downstream pathways: PI3K/AKT/mTOR, RAS/RAF/MEK/ERK1/2 or MAPK, PIP2/DAG/PKC, STAT, p53, and β-catenin pathways - Development - Cell proliferation - Apoptosis regulation - Cell migration - Angiogenesis	FGF19: [308] ↓ gluconeogenesis ↑ glycogen synthesis ↑ peripheral insulin sensitivity ↑ glucose metabolism ↓ lipogenesis ↑ fatty acid oxidation FGF21: [309,310] - ↑ PGC-1α - ↑ mitochondrial ATP production - ↑ hepatic gluconeogenesis - ↑ ketogenesis Mitochondrial FGFR1-like receptor [310]: - ↑ PDHK1 ┤PDH → ↓ pyruvate to acetyl-CoA conversion → ↓ glycolysis α/βKlotho (tumor suppressor effects—some effects only seen in tumor cells) [311,312,313]: ↓ glycolysis (via HK, PFK-1, PK2, PDHK1) ↓ fatty acid synthesis ↓ GLUT expression (GLUT1, GLUT4) ↓ lactate transporter expression (MCT4) ↓ ROS production ┤autophagy
*TP53* /p53 Tumor suppressor	Wild-type TP53: - Cell cycle arrest - Growth arrest - DNA repair - ↑ senescence	Wild-type TP53: [1] - ↑ apoptosis (↓ Bcl2 and ↓ BclXl; ↑ Bax and ↑ Bak), - ↑ glycolysis - ↑ OXPHOS - ┤ pentose phosphate pathway - ↓ glucose receptor expression (GLUT1, GLUT3, GLUT4) - ┤ fatty acid synthesis - ┤ mitochondrial fission (via Drp1) - ↑ autophagy (via mTOR-dependent and independent manner) - ↓ ROS production
*ARID1A* Tumor suppressor	- Cellular differentiation - Cell cycle regulation - Cell migration - Angiogenesis - DNA repair For downstream pathways, see Figure 4, Figure 5 and Figure 6	ARID1A loss: - ↑ mitochondrial membrane potential [391] - ↑ OXPHOS [2,391,392,393] - ↑ anaerobic glycolysis [393] - ↑ mitochondrial mass [391] - ↑ mitochondrial fission [391] For details on its downstream pathways, see Figure 4, Figure 5 and Figure 6
*TERT* Proto-oncogene	Canonical function: - Telomere elongation Noncanonical functions: - Chromatin structure regulation - RNA silencing - Epigenetic changes - Mitochondrial effects - Activation of signaling pathways (i.e., NF-κB and Wnt/β-catenin signaling pathways) - ↑ cell adhesion and migration	TERT expression/overexpression: - ↑ or ↓ apoptosis [430] - Directly binds to mtDNA and protects it from ROS-induced damage [433] - ↑ expression of glycolysis enzymes [434] - ↑ glucose flux via the pentose phosphate pathway, ↑ NADPH [435] - ↑ glutathione levels [436] - ↑ mitochondrial mass [434] Loss of mitochondrial TERT: - ↑ autophagy [436] Telomere dysfunction: - ↓ PGC-1α and PGC-1β promoters (decreasing mitochondrial biogenesis) [432]

Abbreviations: AKT: Ak strain transforming/protein kinase B; ARID1A: AT-Rich Interaction Domain 1A; Drp1: dynamin-related protein 1; FASN: fatty acid synthase; FGF: fibroblast growth factor; FGFR: FGF receptor; G6PD: glucose-6-phosphate-dehydrogenase; GLUT: glucose transporter; HK: hexokinase; JUN: JUN N-terminal kinase; LDH-A: lactate dehydrogenase A; MAPK: mitogen-activated protein kinase; MCT1: monocarboxylate transporter 1; Mfn2: mitofusin 2; mTOR: mechanistic target of rapamycin; NF-κB: nuclear factor κ-light-chain-enhancer of activated B cells; OXPHOS: oxidative phosphorylation; PDHK1: pyruvate dehydrogenase (PDH) kinase 1; PFK-1: phosphofructokinase-1; PGC-1: peroxisome proliferator-activated receptor-gamma coactivator-1; PINK1: PTEN-induced putative kinase 1; PTEN-Induced Kinase 1; PI3K: phosphatidylinositol-3-kinase; PK2: pyruvate kinase 2; PTEN: phosphatase and tensin homolog; Ral (RAS-like) small GTPase; ROS: reactive oxygen species; SREBP: sterol regulatory element-binding protein; TERT: telomerase reverse transcriptase; TLR4: Toll-like receptor 4; Wnt: Wingless/int1. ↑: increased, activated and/or induced; ↓: decreased; ┤: inhibition/inhibited.

**Table 5:** Fibroblast growth factor (FGF) proteins and their alterations in breast (BCs), endometrial (ECs), and ovarian cancers (OCs): data collected mostly from the Human Protein Atlas database [291] and from an article by Li, 2019 [292], unless other references are noted.

FGF Subfamily	FGFs	Additional Information	FGF Associations with BC, EC, or OC (Presence of Immunoreactivity (IR) [291] or Increased *FGF* Gene Expression/Activating Mutation/Gene Amplification [291,292])
1	1, 2	“Paracrine” FGFs; Bind to FGFRs via HSPG	1: OC (gene amplification)
4	4, 5, 6	“Paracrine” FGFs; Bind to FGFRs via HSPG	4: BC, EC (both: rare, IR; BC: + gene amplification)
7	3, 7, 10, 22	“Paracrine” FGFs; Bind to FGFRs via HSPG	3: BC (rare: IR; + gene amplification) 7: EC (rare, weak IR) 10: BC (gene overexpression)
8	8, 17, 18	“Paracrine” FGFs; Bind to FGFRs via HSPG	8: EC (increased RNA expression) 17: BC, OC (both weak staining, OC: rare) 18: BC (rare)
9	9, 16, 20	“Paracrine” FGFs; Bind to FGFRs via HSPG	9: EC, OC (both: IR; EC: + gene mutation) 16: OC (gene overexpression) 20: EC (increased RNA expression)
19	19, 21, 23	“Endocrine” FGFs; Bind to FGFRs via α/or βKlotho proteins (obligatory co-receptors)	19: OC, EC (EC: rare and weak staining) αKlotho: tumor suppressor in BC, and OC [293,294] βKlotho: tumor suppressor in EC
11	11, 12, 13, 14	“Paracrine” FGFs; Bind to FGFRs via HSPG; intracellular localization and binding is typical [295,296,297]	-

Abbreviations: BC: breast cancer; EC: endometrial cancer; FGF: fibroblast growth factor; FGFR: FGF receptor; HSPG: heparan sulfate proteoglycan; IR: immunoreactivity; OC: ovarian cancer.

**Table 6:** BAF subunits: their alternative names and their functions [378,379].

Function	Subunits/Alternative Names
Catalytic ATP-ases	SMARCA2/BRM SMARCA4/BAF250B/BRG1
Core subunits	SMARCB1/SNF5/INI1 SMARCC1/BAF155 SMARCC2/BAF170
Signature subunits (BAF)	ARID1A/SMARCCF1/BAF250A ARID1B/BAF250B
Signature subunits (PBAF)	ARID2/BAF200
Accessory subunits	ACTL6A, or B/BAF53A, or B SMARCD1, 2, or 3/BAF60A, B, or C SMARCE1/BAF57 DPF1,2, or 3/BAF45B, C, or D PHF10/BAF45A BRD7, or 9 BCL11A, or B BCL7A, B, or C SS18

Abbreviations: BAF: BRG1-associated factor; SMARCB1: SWItch Sucrose Non-Fermentable (SWI/SNF)-related, Matrix-associated, Actin-dependent Regulator of Chromatin; SWI/SNF: SWItch Sucrose Non-Fermentable.

An in vitro BC study showed that transfection with a TERT-promoter construct resulted in enlarged stem-cell-like cells that showed increased mitochondrial biogenesis and functional activity and increased expression of glycolytic enzymes [434]. A metabolic CNS imaging study showed that in low-grade gliomas, TERT expression increased glucose flux via the pentose phosphate pathway, and it increased NADPH and glutathione levels [435]. In addition, the loss of mitochondrial TERT was shown to induce autophagy [436]. The general and mitochondrial effects of TERT are summarized in Table 4.

*TERT and telomerase function.* The prevalence of upregulated TERT expression and thus telomerase activity in cancers, as well as tumor cell dependence on telomerase function for perpetual replication, makes telomerase a valuable target for cancer therapy. Current therapeutic strategies for targeting telomerase include immunotherapies, direct small-molecule inhibitors, altered *TERT* gene expression, and the indirect disruption of telomerase regulation [437]. A possible limitation to targeted telomerase therapy is, however, the time required for telomerase inhibitors to impact telomere length [438]. This may require long periods of treatment and increase the risk of resistant clones. Targeting mitochondrial dysfunction in *TERT*-altered tumors, therefore, might be beneficial.

## 5. Similarities and Differences between BCs, ECs, and OCs

When looking at similarities among the three tumors discussed, there definitely is a significant overlap in the genetic alterations associated with them. Additionally, all three of them generally have increased anaerobic glycolytic activity, with variable OXPHOS capacity. In BC, OXPHOS is typically decreased, with more aggressive tumors showing the most severe defects (see Section 2.1). EC, on the other hand, is typically associated with increased OXPHOS, especially *TP53*-altered tumors, with reduced OXPHOS also described in type I ECs, likely contributing to their less aggressive nature (see Section 3.1). In OCs, OXPHOS is variable (see Section 4.1). In addition, mitochondrial fission and Drp1 expression are increased in BC and OC, with an increased mitochondrial mass in BCs, ECs, and OCs. Furthermore, somatic and germline mtDNA mutations (D-loop and other areas) are more frequently recognized in all three tumor types, along with nuclear DNA mutations affecting mitochondria (see individual sections for more details).

Furthermore, certain genetic alterations may change the metabolic profile and mitochondrial function of any of these three tumors. The presence of PI3K/AKT/mTOR pathway activation, for example, could result in the inhibition of the pentose phosphate pathway or glycolysis. Another example would be the presence of FGFR alterations, where increased FGF19 may decrease lipogenesis and glyconeogenesis or where increased FGFR1-like receptor expression would inhibit glycolysis.

Another common participant in the pathogenesis of type I ECs, OCs, and BC is estrogen. Interestingly, previous reports described its direct mtDNA binding via the ERα and ERβ receptors [439]. In addition, it has an anti-apoptotic effect via Bcl-2/Bcl-Xl upregulation and Bax downregulation [6,440,441]. Moreover, estrogen alters the mitochondrial fission/fusion ratio, resulting in an increased fusion tendency. Estrogen treatment also induces mitochondrial biogenesis with increased TFAM and PGC-1α expression [6,442,443,444]. In addition, a recent article reported that an estrogen-responsive gene, cytochrome c oxidase (COX) subunit 7a-related polypeptide (*COX7RP*, also known as *COXA2L* or *SCAF1*), is highly expressed in both BC and estrogen-driven ECs, increasing their hypoxia tolerance. COX7RP works as a promoting factor for mitochondrial respiratory supercomplex assembly, leading to efficient OXPHOS. COX7RP overexpression, associated with an inferior prognosis in BC patients, promoted EC growth in an in vivo model and alters the metabolic profile of cancer cells. It largely affects glucose homeostasis and regulates the expression of TCA intermediates [445].

## 6. Conclusions

Are mitochondrial-targeted therapies going to be used in BC, EC, or OC? Based on the evidence that, in many cases, therapy-resistant tumors have significant, targetable metabolic and/or mitochondrial changes, it is definitely promising. In addition, several drugs were shown to be useful in preclinical and/or clinical settings—some of them in other cancer types. Having information on the genetic changes in a tumor sample may help to individualize metabolic targeting.

## Figures and Tables

**Table 1:** Clinicopathological subtypes of BC (adapted from [19,20]), heavily relying on hormone receptor expression status and cell proliferation index (Ki-67) to predict clinical behavior and therapeutic response to certain drugs.

Subtype	Clinicopathological Definition
Luminal A	“Luminal A like” ER-positive HER2-negative Ki67 low PR high
Luminal B	“Luminal B-like (HER2-negative)” ER-positive HER2-negative and either Ki67 high or PR low “Luminal B-like (HER2-positive)” ER-positive HER2-positive Any Ki67 Any PR
HER2 positive	“HER2-positive (non-luminal)” HER2-positive ER and PR absent
Triple negative	“Triple-negative” ER and PR absent HER2-negative

Abbreviations: ER: estrogen receptor; HER2: human epidermal growth factor receptor 2; PR: progesterone receptor.

**Table 2:** Role of metabolic changes in breast cancer.

Metabolic Pathway	Enzyme/Protein	Role in Breast Cancer
*Anaerobic glycolysis*	*Enolases*	α-Enolase gene expression correlates with tumor size and shorter disease-free interval [25,26]
	*Pyruvate kinase*	Levels elevated in aggressive breast cancer type [28]
*Oxidative phosphorylation* *(OXPHOS)*	*Complex I, II, III, and IV*	Aggressive breast cancer shows the broadest OXPHOS defect in cell lines [29]
	*UQCRFS1 and UQCRH (complex III subunits)*	Increased expression in breast tumors compared to normal breast tissue [29]
*Other metabolic markers*	*Hydratases*	MTHFD2 protein content 3-fold decreased in breast cancer cell line [31]
	*Dehydrogenases*	IDH2 expression elevated in breast cancer cell lines. Expression is positively associated with overall survival [32].
	*Oxidases*	Coproporphyrinogen III oxidase expression elevated in Adriamycin-resistant breast cancer cell lines [33]

## Data Availability

Not applicable.
